# Machine Learning, Deep Learning, and IoT for Waste Classification and Intelligent Waste Management: A PRISMA-Based Systematic Review

**DOI:** 10.3390/s26206569

**Published:** 2026-10-16

**Authors:** Md. Rahat Khan, Md. Abdus Salam, Farhana Akter, Md. Wahidur Rahman, Haitham Y. Adarbah, Tarek Mahmud, Afzel Noore

**Affiliations:** 1 Department of Computer Science and Engineering, Khwaja Yunus Ali University, Sirajganj 6751, Bangladesh; rahat.cse@kyau.edu.bd (M.R.K.); salamshomaji@gmail.com (M.A.S.); akterbonna0209@gmail.com (F.A.); 2 Department of Electrical Engineering and Computer Science, Texas A&M University-Kingsville, Kingsville, TX 78363, USA; tarek.mahmud@tamuk.edu (T.M.); afzel.noore@tamuk.edu (A.N.)

**Keywords:** waste classification, intelligent waste management, convolutional neural networks, deep learning, Internet of Things, PRISMA, systematic literature review, federated learning, deployment readiness, waste management research trends

## Abstract

The rapid growth of urban populations and consumption patterns has significantly increased the volume and complexity of municipal waste streams, including recyclable, organic, hazardous, and electronic waste. Traditional waste management approaches such as manual sorting and fixed collection schedules struggle to handle the diversity and scale of modern waste systems efficiently. Consequently, recent research has explored artificial intelligence (AI), computer vision, and Internet of Things (IoT) technologies to automate waste classification and enable intelligent monitoring and management of waste infrastructures. However, the rapidly expanding literature in this domain is fragmented across different datasets, sensing platforms, and experimental settings, making it difficult to obtain a comprehensive understanding of current technological progress. To address this gap, this study conducts a systematic literature review using the PRISMA methodology, analyzing 50 rigorously selected peer-reviewed studies on automated waste classification and intelligent waste management systems. The protocol was registered in the Open Science Framework (OSF). The reviewed works are examined in terms of model architectures, sensing infrastructures, datasets, and system capabilities. The analysis shows that convolutional neural network architectures such as ResNet, MobileNet, and EfficientNet, together with object detection frameworks including YOLO and Faster R-CNN, dominate current visual waste classification research; however, emerging studies increasingly explore hybrid architectures combining convolutional networks with transformer-based models to improve contextual understanding and scalability. IoT-enabled smart bins equipped with ultrasonic sensors, load cells, and environmental sensors are widely used for real-time waste monitoring. The review also reveals a recent surge of research activity, particularly in Asia, with increasing contributions from major publishers such as Elsevier and MDPI. Despite promising experimental performance, most systems remain limited to small curated datasets and pilot-scale deployments, highlighting a gap between laboratory research and real-world implementation. These findings provide a consolidated overview of current research trends and identify key challenges for developing scalable and practical intelligent waste management systems.

## 1. Introduction

In recent decades, waste management has escalated into one of the most pressing global environmental and socioeconomic crises. This crisis is fueled by a perfect storm of rapid urbanization, aggressive industrial expansion, and increasingly unsustainable consumer consumption patterns [1]. This trajectory is not inherently inevitable; where collection and treatment capacity expands in step with waste volume, adverse outcomes can be substantially mitigated [2]. In practice, however, infrastructure investment has consistently lagged behind waste generation growth, particularly in rapidly urbanizing regions, turning a manageable scaling problem into a compounding crisis [2]. According to the United Nations Environment Programme (UNEP) 2024 report, the world generated approximately 2.1 billion tons of municipal solid waste (MSW) in 2023 [2]. Without immediate systemic intervention, this volume is projected to surge by more than 60%, reaching a staggering 3.8 billion tons by 2050 [3]. The complexity of the waste stream is further exacerbated by the rise of specialized materials. For instance, the Global E-waste Monitor 2024 indicates that electronic waste reached a record 62 million tons in 2022, growing five times faster than documented recycling efforts [4]. These mounting volumes impose severe pressures on global ecosystems, with improper management responsible for roughly 20% of global human-related methane emissions, a potent driver of climate change [5].

Developing nations remain the most vulnerable to these shifts; current data suggests that in low-income regions, over 90% of waste is still openly dumped or improperly managed due to a critical lack of classification and treatment infrastructure [2]. The human cost of this gap is well documented: the 2017 collapse of the Koshe landfill in Addis Ababa, Ethiopia, buried a nearby settlement and killed more than 60 people [6]; similarly, Uncontrolled landfill fires caused by methane buildup and leachate contamination at sites such as the Bantargebang landfill in Indonesia have repeatedly compromised local water supplies and air quality [7]. Such incidents illustrate that inadequate waste segregation and treatment translate directly into loss of life, environmental contamination, and public-health burden, rather than remaining an abstract statistical concern. Addressing this gap requires waste to first be reliably separated into recyclable, organic, hazardous, and residual streams, a precondition for any downstream treatment, recycling, or safe disposal pathway. Manual sorting, however, does not scale with the volume and heterogeneity of modern waste streams, is labor-intensive, and exposes workers to hazardous or infectious material [8]; fixed-schedule collection similarly cannot adapt to variable fill rates or waste composition. Consequently, the development of automated waste classification systems has transitioned from a technological luxury [9] to a practical necessity for achieving reliable, scalable segregation at the volumes required for a circular economy. In contrast, the integration of artificial intelligence (AI) and machine learning (ML) into waste processing systems appears to vary significantly across different regions of the world. While advancements in DL and computer vision have enabled automated waste classification, the extent and distribution of their implementation remain unclear [10].

Existing reports indicate that advanced waste classification systems are exclusively deployed in high-income regions [11]. Europe and North America have adopted automated sorting technologies in response to circular economy initiatives, Japan has incorporated robotics for high-precision material recovery [12], and selected smart cities in China have implemented AI-assisted sorting infrastructures [2]. However, the global landscape of intelligent waste classification adoption has not been systematically synthesized. Traditional waste management systems primarily rely on manual sorting, fixed collection schedules, and landfill-based disposal practices [11]. These approaches present operational and environmental challenges, particularly in rapidly urbanizing and densely populated regions. Issues such as landfill overflow, greenhouse gas emissions, and inefficient resource recovery have increased the need for more adaptive and technology-driven waste management solutions.

**Figure 1:**
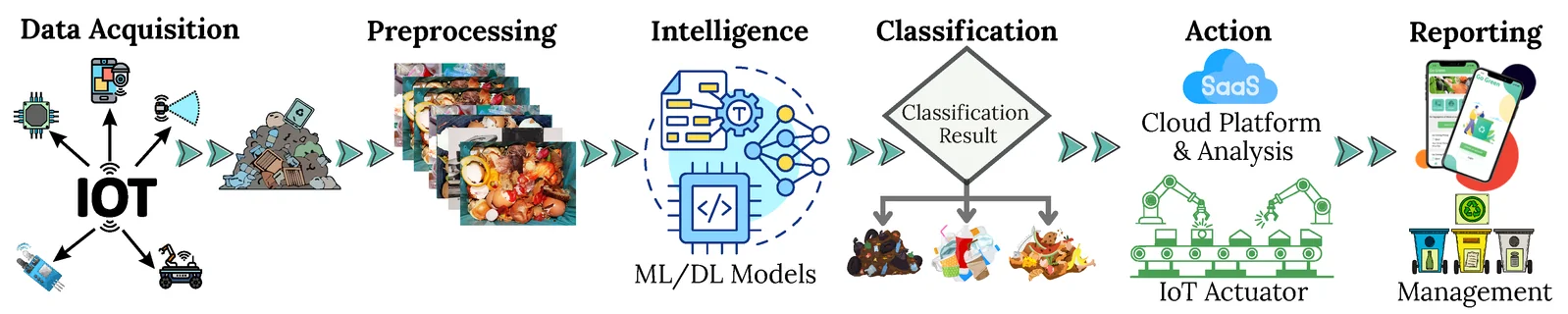
Conceptual flow of an IoT-enabled smart waste classification framework integrating sensor-based data acquisition, IoT connectivity, preprocessing, and machine learning and deep learning techniques for automated waste identification, classification, monitoring, and intelligent waste management.

In response, smart waste management frameworks integrating Internet of Things (IoT) technologies and AI have gained growing research attention. IoT-enabled systems facilitate real-time monitoring of waste bins, environmental conditions, and collection logistics through interconnected sensing and communication infrastructures [13]. When combined with machine learning techniques, these systems support data-driven decision-making for routing optimization, anomaly detection, and operational planning. To illustrate the operational architecture of such solutions, Figure 1 depicts a conceptual framework for an IoT-enabled smart waste classification system, demonstrating the end-to-end data flow from edge perception to AI-driven processing and final actuation.

Recent research has explored deep learning–based computer vision models for automated waste classification. Convolutional neural networks (CNNs) and object detection architectures such as ResNet, EfficientNet, MobileNet, and YOLO have been applied to categorize recyclable, organic, hazardous, and mixed waste streams [13,14,15,16,17,18]. Additionally, predictive modeling approaches including recurrent neural networks and spatiotemporal learning methods have been investigated for forecasting waste generation patterns and bin fill levels within urban environments [19,20,21,22].

Although a diverse range of technological approaches has been proposed across different application settings, the overall research landscape remains distributed across multiple domains, datasets, and deployment contexts. A structured synthesis is therefore required to systematically examine methodological trends, technological choices, and implementation characteristics within AI-driven waste classification research.

To provide a structured synthesis of recent advancements, this study conducts a systematic literature review (SLR) of 50 peer-reviewed articles with unbiased, systematic procedures published between 2020 and 2025 (42 identified via primary database search; 8 via citation-tracking and a 2026 search update). Most of the selected studies were drawn from high-impact journals and represent contributions from multiple countries and publishers. The review systematically analyzes the literature with respect to model architectures, datasets, hardware configurations, system-level integration, usability considerations, reported limitations, and environmental implications. Through this synthesis, the study aims to provide structured insights to support the development of scalable, deployable, and environmentally responsible waste management systems. The primary contributions of this review are summarized as follows:

- This review conducts a systematic review of 50 peer-reviewed studies using the PRISMA framework to ensure transparent, structured, and reproducible selection and analysis, directly supporting the evidence base required for RQ1 and RQ2.
- It systematizes the literature into four technical clusters: IoT architectures, deep learning recognition, predictive analytics, and emerging frontiers to provide a structured evaluation of the waste classification and intelligent management landscape.
- It performs a comparative evaluation of deep learning architectures, performance metrics, dataset characteristics, hardware configurations, and system usability to identify methodological trends, addressing RQ1.
- It examines the integration of AI with IoT infrastructures (UAVs, smart bins) and synthesizes their environmental benefits regarding efficiency and emissions.
- It consolidates commonly reported practical limitations, such as restricted dataset diversity, environmental condition sensitivity, limited multimodal validation, and constrained large-scale real-world deployment, addressing RQ2’s deployment-readiness assessment.
- It identifies emerging directions (edge AI, blockchain, federated learning) and outlines a future pipeline for resilient waste management systems.

Unlike prior reviews that isolate a single analytical layer model taxonomy alone [10], municipal financing [23], narrative AI + IoT overviews without systematic protocol [24], broad municipal AI scope without classification specificity [25], LCA only [26], or ML/DL without IoT integration [27,28], this review’s novelty is operationalized as five auditable analytical dimensions applied uniformly across all 50 included studies: (i) model architecture family and its reported headline metric (Section 3.1.1); (ii) sensor/hardware configuration and IoT communication stack (Section 3.2.1); (iii) deployment-readiness stage, scored against the explicit RQ2 framework simulation, laboratory prototype, pilot deployment, or multi-site deployment (Section 2.1 and Section 3.1.2); (iv) degree of cross-layer integration between perception, sensing, and predictive/optimization modules (Section 3.2); and (v) reported (vs. merely claimed) environmental/sustainability impact (Section 3.2.4). Because each dimension is extracted per study and tabulated, the resulting synthesis is directly checkable against the source studies, rather than a narrative claim of comprehensiveness.

To systematically present these insights and the underlying evidence, the article is structured as follows. Section 2 explains the methodology used to conduct the systematic literature review, including data sources, selection criteria, and quality assessment procedures. Section 3 presents the results with key findings, organized around thematic clusters, highlighting important patterns, observations, and research gaps. Section 4 summarizes constructive comments provided by the authors, and outlines limitations identified in the reviewed studies. Finally, Section 5 concludes the paper with suggested future research directions and practical recommendations based on the synthesized insights.

## 2. Methods

This review follows a structured, three-stage process (Planning, Conducting, and Reporting) designed to ensure the results are both transparent and reproducible. To maintain high methodological rigor and standards, we adopted the PRISMA 2020 guidelines [29]. The review protocol was registered in the Open Science Framework (OSF; https://doi.org/10.17605/OSF.IO/G9JEK). This framework acts as a pipeline, guiding the study from the initial literature identification through to the final qualitative synthesis. Our process began with a strict ‘Protocol Definition’ phase, where we established the scope of our inquiry into AI, ML, DL, and IoT applications within waste management.

A critical first step in this planning stage is narrowing our focus to the specific problems we aim to solve. This leads directly into the formulation of our research questions.

### 2.1. Research Question

We formulated our research questions using the PICOC framework (Population, Intervention, Comparison, Outcome, and Context). This structure is particularly effective for reviews that address public issues, as it ensures the technology is evaluated not just on theoretical performance, but on its practical application in real-world contexts.

To capture both the technical performance and the operational maturity of current solutions, we developed the following two research questions:

RQ1: Reported Technical Performance: What is the reported technical performance of current AI and IoT-driven architectures across diverse waste management domains, and, where a traditional/non-AI baseline is reported in the primary study, how does performance compare to that baseline?
  Motivation: This question quantifies the efficiency and accuracy of AI/IoT interventions across different waste populations (e.g., solid, hazardous, e-waste), and characterizes rather than assumes the evidence base for superiority over traditional methods, since only a subset of included studies report a traditional method comparator directly.
RQ2: Scalability and Deployment Readiness: To what degree are these solutions prepared for real-world scalability and operational deployment, assessed against a predefined deployment-readiness framework, and what input data/variable types underpin this readiness?
  Motivation: This question addresses the scalability gap using an explicit, predefined set of readiness criteria validation setting (simulation/laboratory prototype/pilot deployment/multi-site deployment), hardware validation, latency, operating duration, communication reliability, environmental robustness, and the modality and source of input data relied upon (e.g., RGB imagery, ultrasonic/weight/gas sensor streams, socioeconomic or chemical-composition variables), rather than an unstructured judgment of “readiness.” Data modality is treated here as a deployment-readiness attribute rather than a separate research question, since the practicality of scaling a system is directly constrained by the availability, cost, and reliability of the data type it depends on (e.g., an image-only classifier scales differently than one requiring lab-measured chemical assays)

### 2.2. Search and Selection Strategy

To effectively address the research questions, we translated the key components of the PICOC framework into specific search keywords. Our goal was to capture the intersection of advanced computational techniques (deep learning/AI) and physical infrastructure (IoT) within the waste management domain. We constructed three primary search strings to ensure broad coverage of both established terminologies and emerging hybrid technologies. Boolean operators (AND, OR) were used to combine these terms effectively.

The search strings were categorized as follows:

- Population and Context: (“Waste management” OR “Solid waste management” OR “Municipal solid waste” OR “Smart waste management” OR “Recycling automation” OR “Waste sorting” OR “Robotic waste sorting” OR “AI waste classification”)
- Intervention: (“Deep Learning” OR “Convolutional Neural Networks” OR “CNN” OR “Reinforcement Learning” OR “Transfer Learning”) AND (“Internet of Things” OR “IoT” OR “Smart Bins” OR “Sensors”)
- Outcome: (“Deep learning waste sorting” OR “Hybrid deep learning” OR “CNN transformer fusion” OR “waste image classification” OR “circular economy waste” OR “sustainable waste management”)

We note that the Population/Intervention/Outcome search blocks were weighted toward visual classification and detection terminology, reflecting the initial scoping of the review around perception-layer methods. Studies addressing collection-route optimization, bin-fill forecasting, and non-visual sensor fusion, which form Section 3.2.3 and part of Section 3.2.1 of our thematic synthesis, were substantially captured through the “Internet of Things,” “IoT,” “Smart Bins,” and “Sensors” terms already present in the Intervention block, and through the supplementary Google Scholar citation-tracking step, rather than through dedicated routing/forecasting search terms. We executed these search strings across four major bibliographic databases: Scopus, Web of Science (Core Collection), IEEE Xplore, and PubMed/MEDLINE. These four were selected jointly for their multidisciplinary, cross-publisher coverage (Scopus and Web of Science), their depth in engineering and computer-science literature (IEEE Xplore), and their coverage of environmental-health and biomedical waste literature (PubMed/MEDLINE). Google Scholar was used only as a supplementary citation-tracking tool, to verify that no additional peer-reviewed studies meeting our eligibility criteria had been missed by the four primary databases; it was not used, and should not be read, as a source of grey literature, consistent with our exclusion of non-peer-reviewed material (Section 2.3).

Each database was queried independently using syntax adapted to its own query language, with an identical date filter and a document-type filter restricting results to peer-reviewed journals or conferences, consistent with the document-type filters applied per database. The full, verbatim search string, applied filters, search date, and number of records retrieved for each database are reported in Appendix A. The initial search was run on 18 November 2025 and returned 315 records across the four databases (Scopus n=150, Web of Science Core Collection n=75, IEEE Xplore n=60, PubMed/MEDLINE n=30).

**Figure 2:**
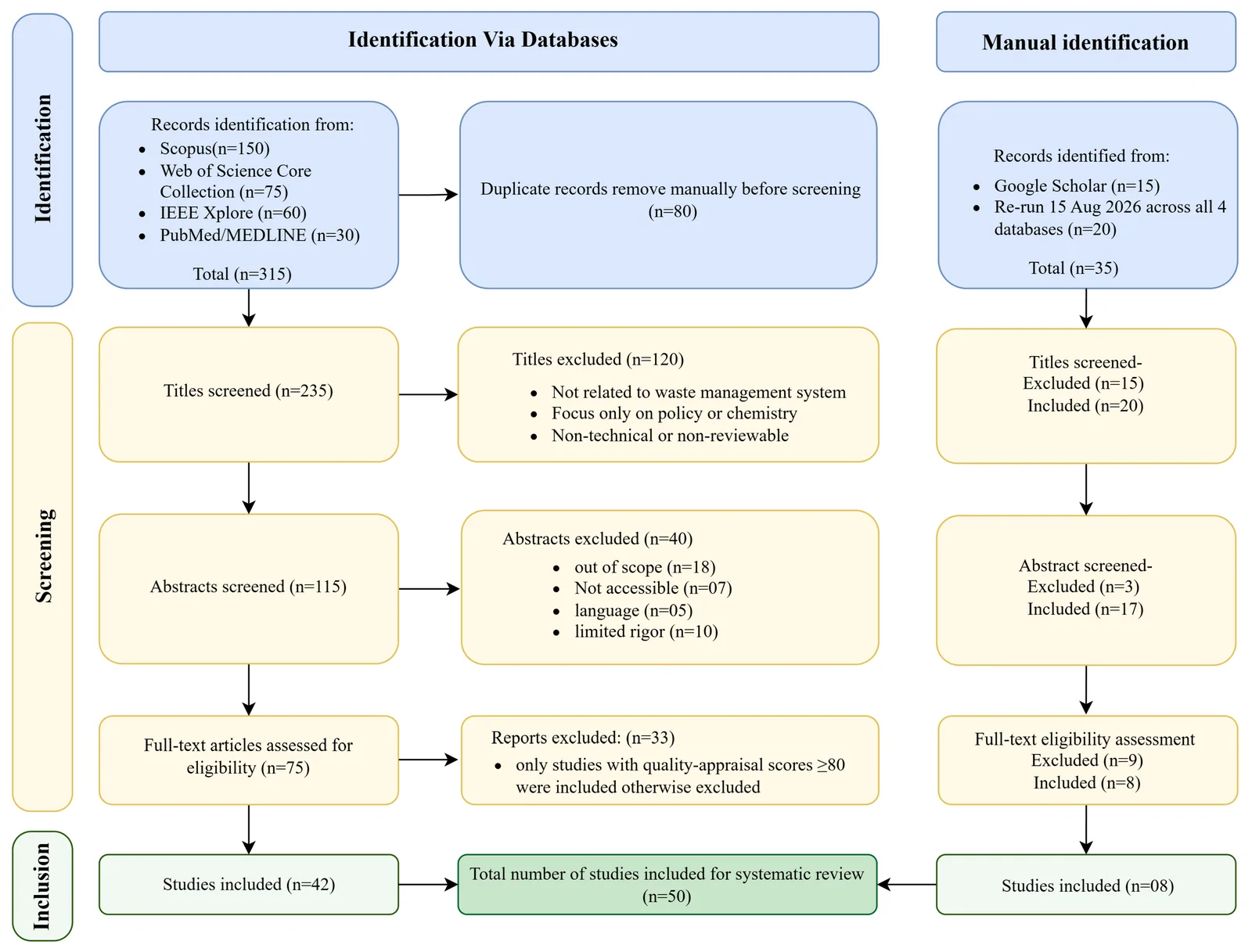
PRISMA 2020 flow diagram detailing the systematic review selection process. The identification phase yielded 315 records from four electronic databases (Scopus, n=150; Web of Science Core Collection, n=75; IEEE Xplore, n=60; PubMed/MEDLINE, n=30), plus 15 additional records identified through manual/citation-tracking (Google Scholar), with 20 additional record added after re-running the search string. Following the removal of 80 duplicates (identified via manual title/author/year matching in a consolidated CSV file), 235 titles were screened; 120 were excluded, leaving 115 abstracts for review, of which 40 were excluded (out of scope, n=18; not accessible, n=7; non-English, n=5; limited methodological rigor, n=10) of 75 full-text articles assessed for eligibility, 33 were excluded because their quality-appraisal score fell below the pre-specified threshold of 80/100, yielding 42 studies from the primary database search. Separately, Google Scholar citation-tracking identified 15 additional records, and a search update run on 15 August 2026 across the same four databases identified a further 20 records (total additional pool, n=35). After title, abstract, and full-text screening against the same eligibility criteria, 8 of these met inclusion requirements (citation-tracking, n=3; 2026 update, n=5). The combined total of 50 studies was included in the final systematic review.

To keep the review current with the submission date, we re-ran the same search strings in all four databases on 15 August 2026; the update search was a supplementary, incremental search, not a re-execution of the full eligibility window restricted to records added to the four databases since November 2025. Publications dated 2026 were not eligible. The update search applied the same publication-year filter (2020–2025) and captured only records from the original eligibility period that had been newly indexed in the four databases between November 2025 and 15 August 2026. The primary search and the supplementary update are reported separately in Figure 2. This update identified 20 additional records, of which 5 met the eligibility criteria and were incorporated into the final set of 50 included studies.

The review window was deliberately bounded to January 2020 onward for two reasons. First, 2020 marks the point at which deep learning architectures like ResNet, EfficientNet, MobileNet, and YOLO-family detectors became the dominant paradigm for image-based waste classification, largely superseding earlier hand-crafted feature and classical machine-learning pipelines; a 2020 start therefore captures the literature most representative of the current technological state addressed by RQ1. Second, low-cost IoT hardware (e.g., ESP32/ESP8266 microcontrollers, Raspberry Pi single-board computers) needed for the smart-bin architectures examined in RQ2 became widely accessible to researchers from approximately 2019 to 2020 onward, meaning earlier studies reflect substantially different hardware baselines with limited relevance to present-day deployment readiness. A six-year window was judged sufficient to both capture this technologically coherent period and yield an adequate volume of eligible studies (315 initial records) for systematic synthesis, consistent with review-window conventions used in other fast-moving AI subfields [30].

### 2.3. Eligibility Criteria

This filtering process prioritized peer-reviewed empirical studies while removing theoretical or non-validated works.

Inclusion Criteria:

- Published as a peer-reviewed journal article or peer-reviewed conference paper, indexed in Scopus, Web of Science, IEEE Xplore, or PubMed/MEDLINE.
- Primary empirical focus on AI, ML, or DL techniques applied to waste management (e.g., classification, sorting, volume estimation, route optimization), with or without IoT-based sensing/data acquisition components.
- Reports a described methodology, dataset, and at least one quantitative performance metric (e.g., accuracy, precision, F1-score, mAP).
- Published between January 2020 and December 2025, identified either through the primary search (run 18 November 2025) or through the supplementary update search (15 August 2026), which captured only records from this period that were newly indexed after the primary search; full text accessible.

For clarity, the technological boundary of this review is defined as follows: studies employing any learned/trained model, including classical supervised ML (e.g., SVM, Random Forest, Decision Trees, gradient boosting) as well as deep learning, were eligible, provided a quantitative performance metric was reported. Studies relying solely on conventional, non-learned image-processing techniques (e.g., fixed-threshold color/shape segmentation, rule-based edge detection, or hand-crafted heuristics without any trained classifier) were considered out of scope and excluded, since such approaches do not constitute AI/ML-driven classification as defined by our PICOC “Intervention” component (Section 2.2). We note that such engineered-feature pipelines remain methodologically distinct from end-to-end deep representation learning: edge, texture, and shape descriptors are designed a priori, whereas CNN/YOLO features are learned from data. An improved cross-algorithm edge detection method for coal gangue identification [31] illustrates this paradigm: edge extraction, morphological segmentation, and mean/contrast/entropy features are engineered, and only the final recognition stage is learned (an SVM). Under our criteria, the edge-detection stage alone would be out of scope, while a pipeline of this hybrid type would be eligible only if applied to waste management with a trained classifier and reported quantitative metrics.

Exclusion Criteria:

- Non-peer-reviewed sources, including preprints, editorials, commentaries, short letters, or purely theoretical/conceptual papers without empirical validation.
- Full text not accessible.
- Studies not published in English.
- Studies outside the scope of intelligent waste management/automation (e.g., general robotics or general IoT papers with no waste-management application).

### 2.4. Data Extraction

**Figure 3:**
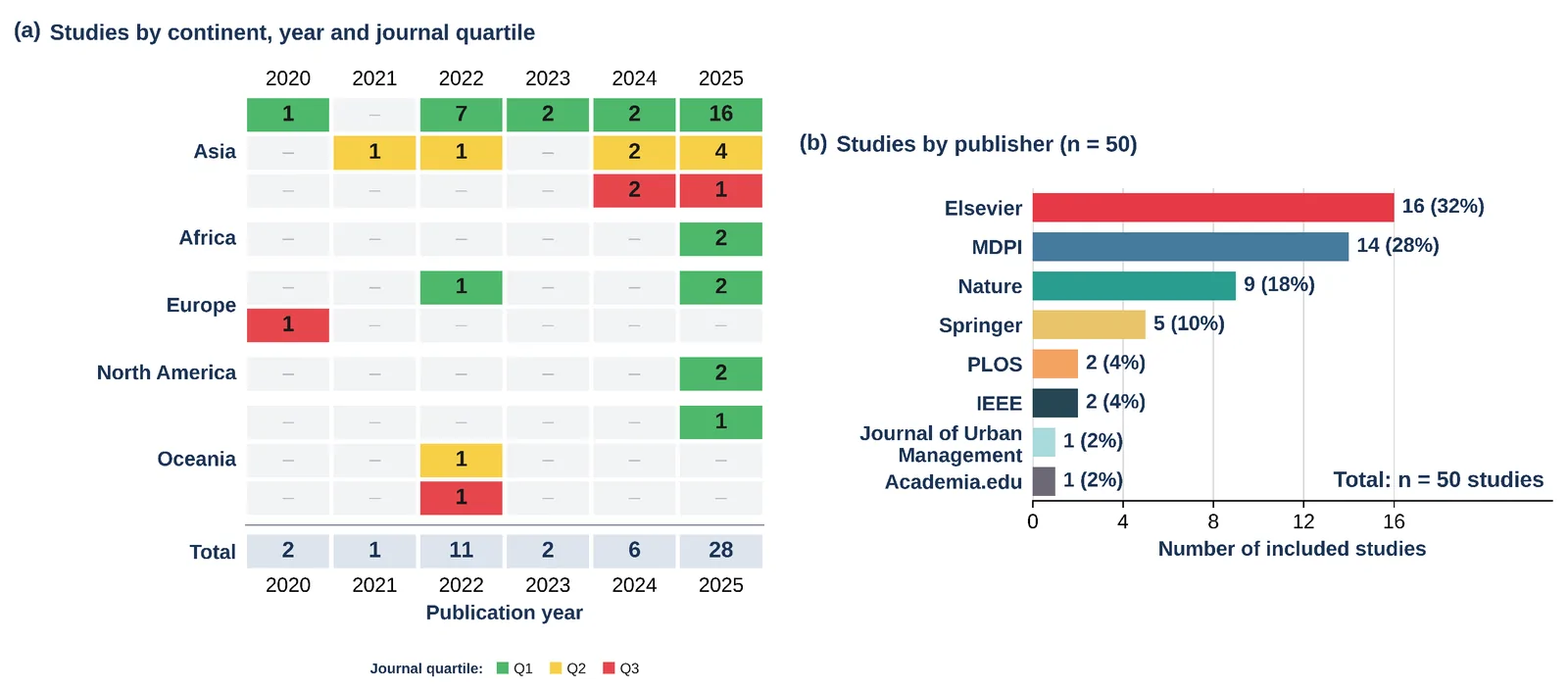
Bibliographic distribution of the included studies. (**a**) Distribution of studies by publication year, continent, and journal quartile (Q1–Q3). The heatmap shows the number of included studies for each continent–year combination, with colors representing journal quartiles. The number of publications increased substantially in 2025, with Asia contributing the largest share of studies, particularly those published in Q1 journals. (**b**) Distribution of the 50 included studies by publisher. Elsevier and MDPI account for the largest proportions of the included publications, followed by Nature and Springer, while the remaining studies are distributed across PLOS, IEEE, Journal of Urban Management, and Academia.edu.

**Figure 4:**
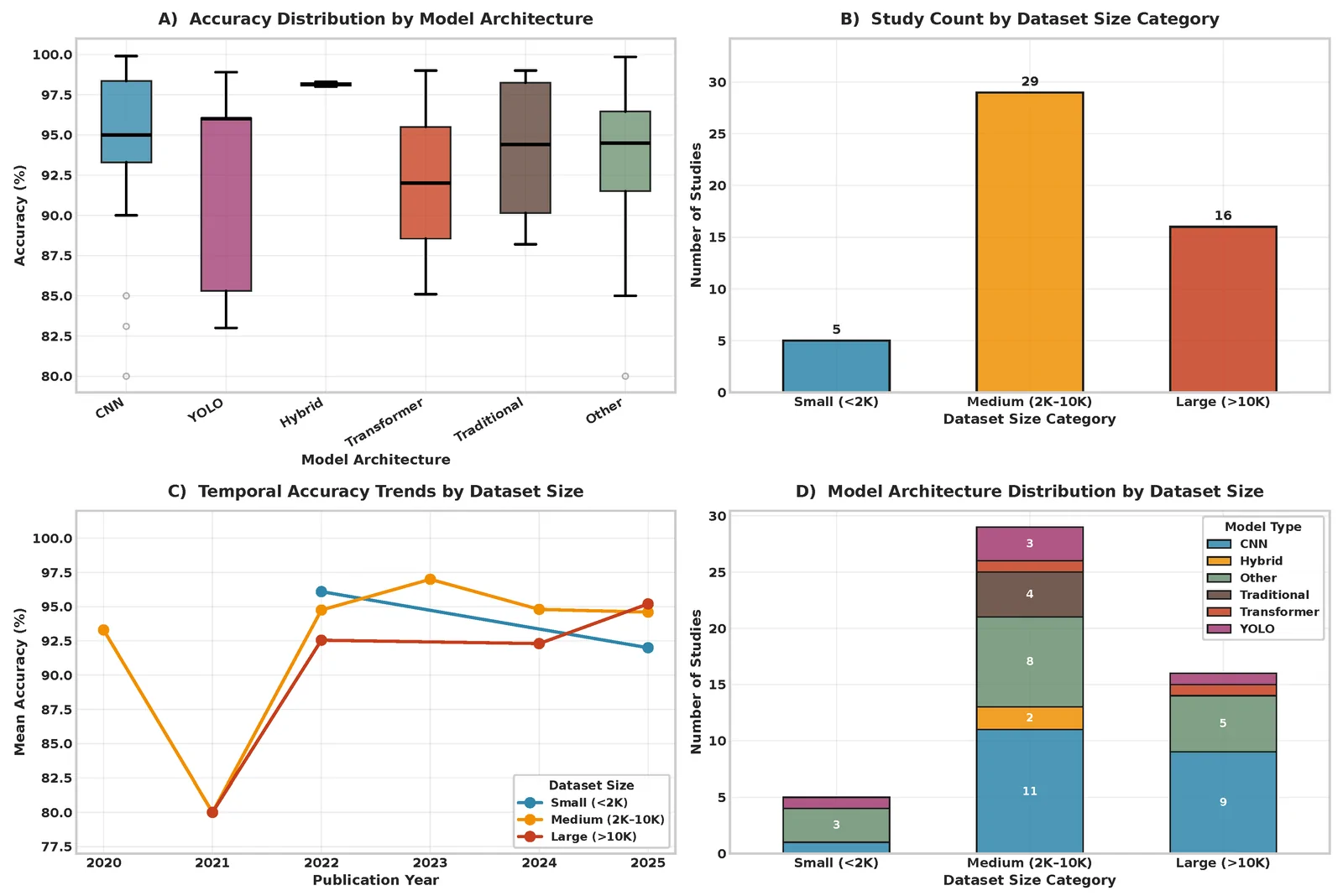
Comprehensive Statistical summary of model performance and dataset characteristics. (**A**) Accuracy distribution by model architecture; (**B**) number of studies by dataset size category; (**C**) temporal accuracy trends by dataset size; (**D**) model architecture distribution by dataset size. Studies whose primary reported outcome is mAP, $R^{2}$, correlation, or MAPE/RMSE are excluded from these accuracy-based visualizations to avoid combining non-commensurable measures.

**Figure 5:**
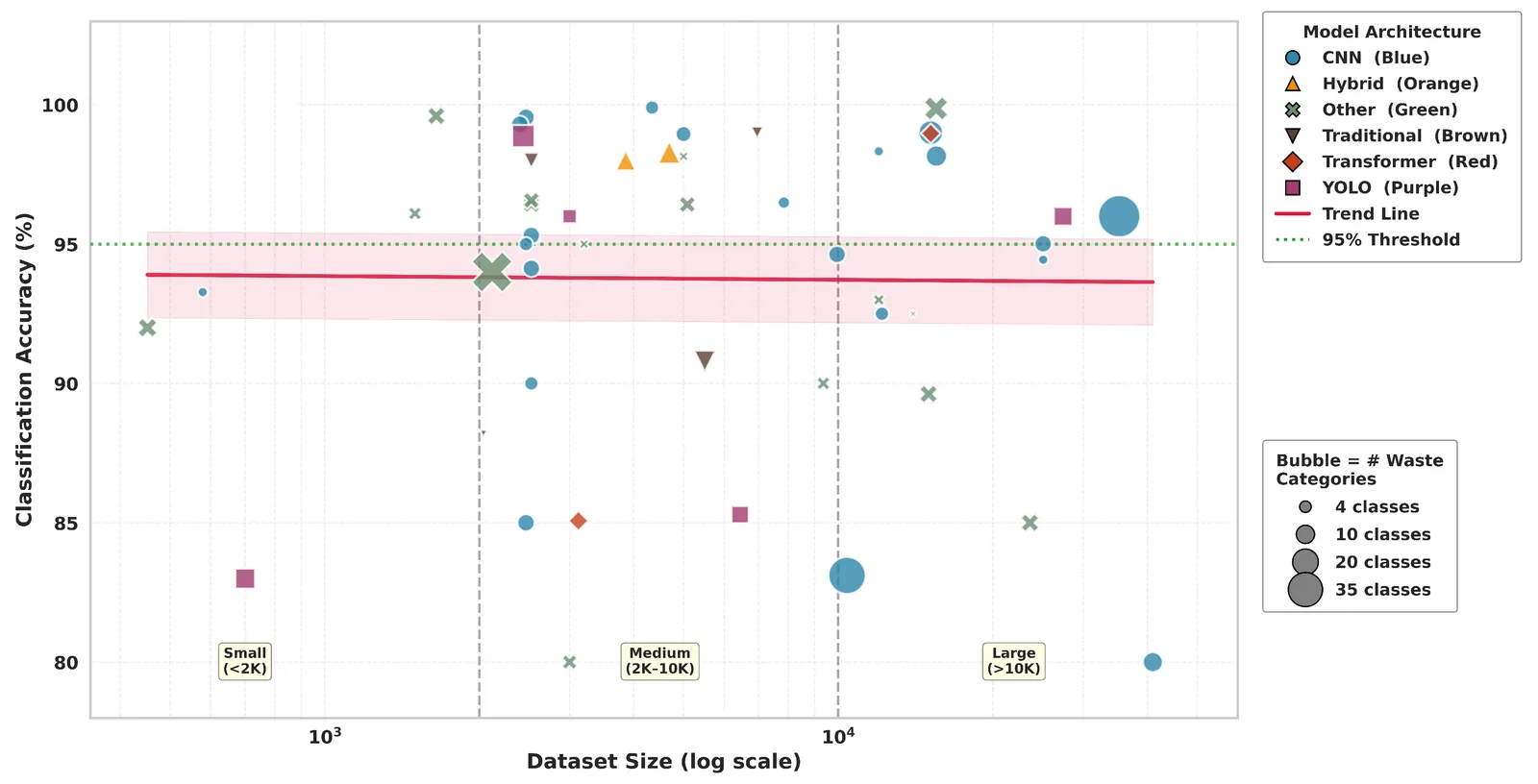
Dataset size vs. accuracy by model architecture. Vertical dashed lines indicate the boundaries between dataset size categories: small (<2K), medium (2K–10K), and large (>10K) images. Plotted values are restricted to the subset of studies reporting classification accuracy as their primary metric; studies whose primary reported outcome is mAP, R^2^, correlation, or MAPE/RMSE are excluded, consistent with the accuracy-only subset used in the DL performance summary (Section 3.1.1).

**Figure 6:**
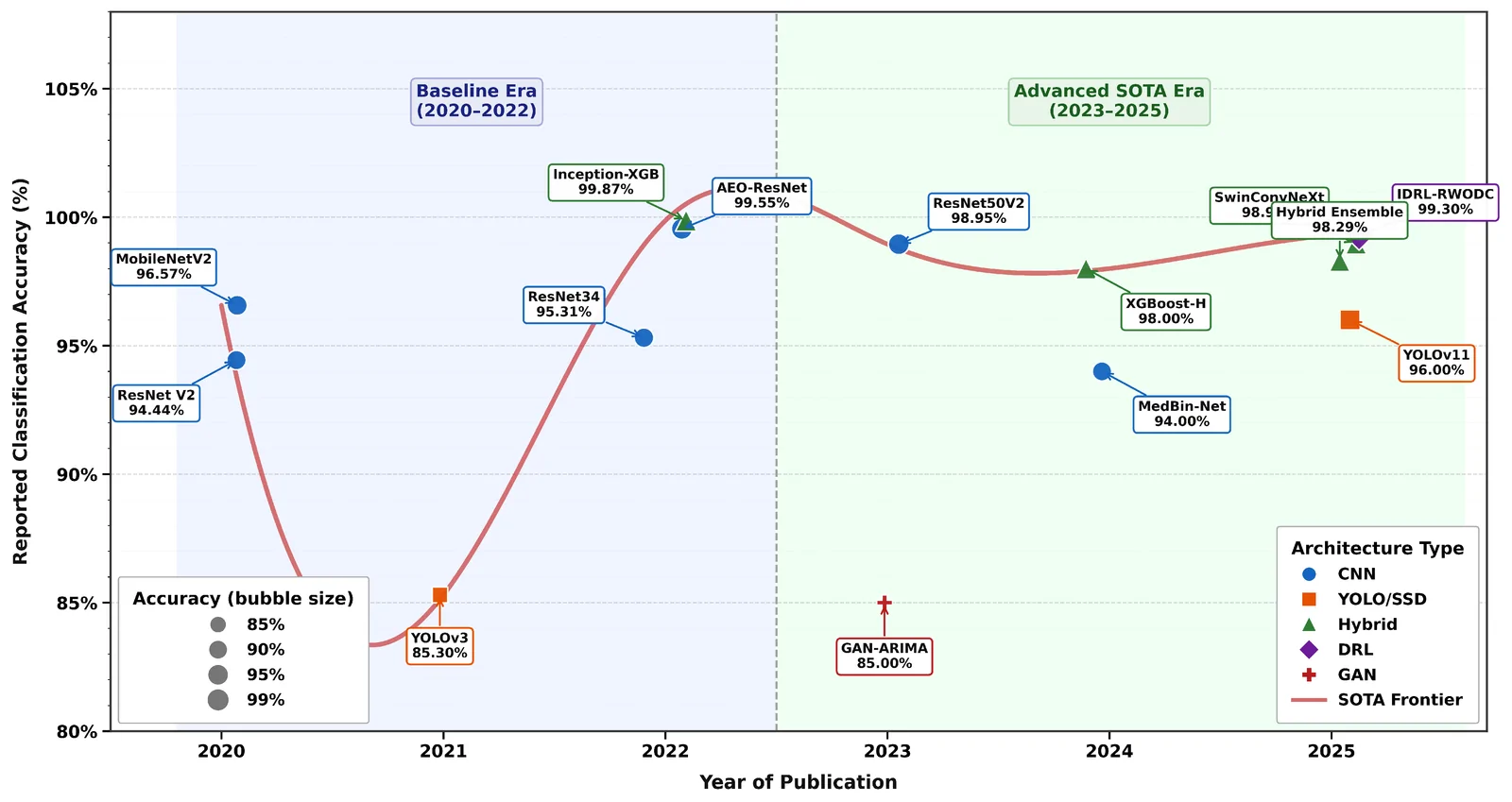
Evolution of state-of-the-art (SOTA) technological frontiers in intelligent waste management from 2020 to 2025, vertical dashed lines indicate the boundaries between publication years highlighting the progression from conventional AI-based waste classification toward advanced edge AI, IoT-enabled monitoring, UAV-assisted detection, multimodal learning, federated learning, and domain-specific intelligent waste management solutions. Each point reflects the reported headline performance value for that study; where this value is not classification accuracy (e.g., mAP, F1-score), it is plotted on the same axis for chronological/SOTA-trend comparison only and should not be interpreted as an accuracy figure or pooled with true accuracy values.

**Figure 7:**
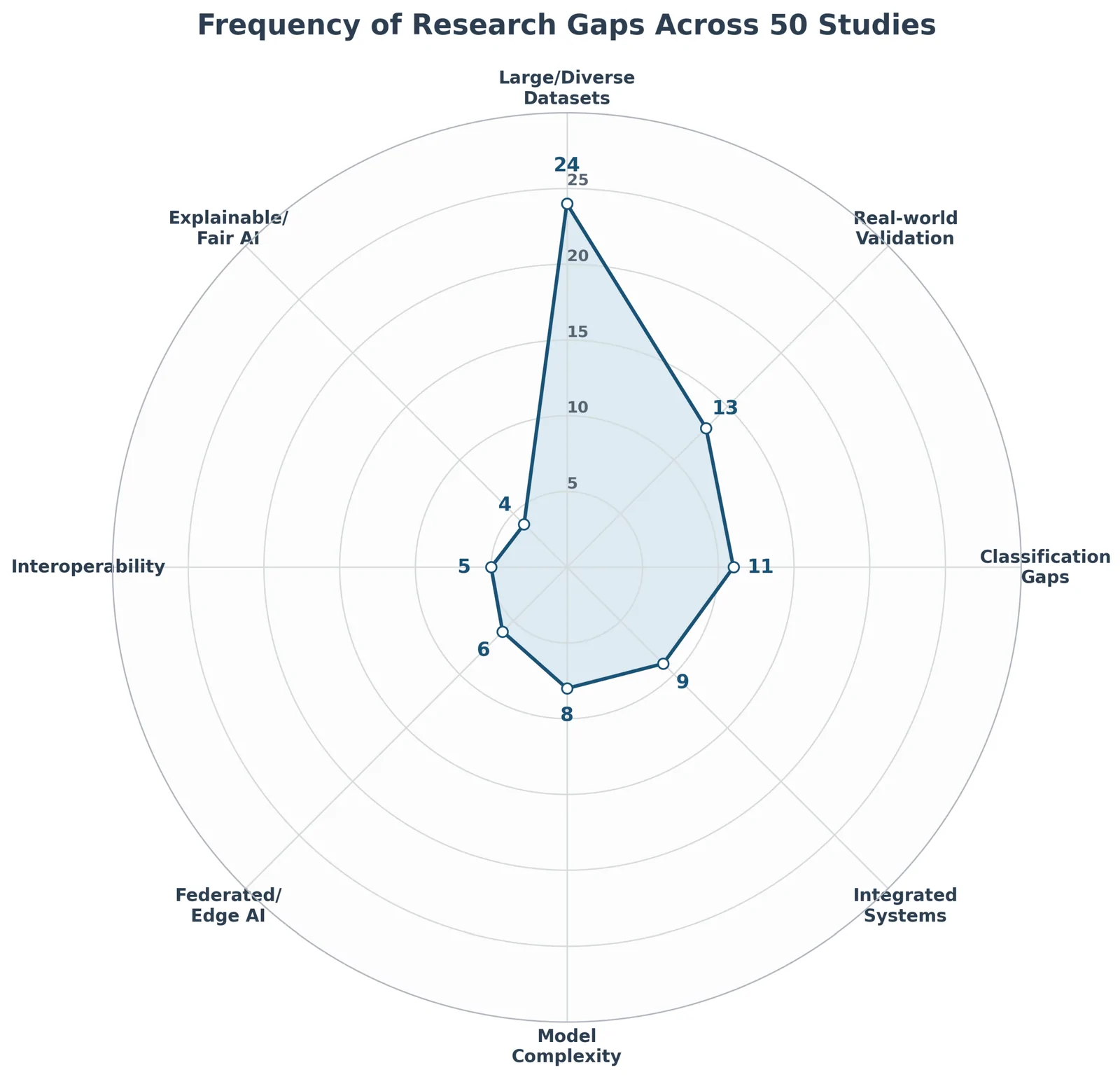
Spider chart of research-gap frequencies across the 50 reviewed studies. Gap categories were identified manually by the authors through thematic reading of the free-text “Limitations” field extracted for each study metadata analysis (summarized in Section 3.1), from which the eight most frequently recurring limitation themes were identified and quantified as shown. Coding was non-mutually-exclusive: a study could be tagged under more than one category where its authors explicitly reported limitations spanning multiple themes, so the category counts shown sum to more than 50.

Data from each of the 50 included studies were extracted by the authors using a standardized single-study extraction template (approximately 1–2 pages per study), applied uniformly across the dataset. Each template captured the following fields: Paper No., Paper Title, Authors, Country, Year, Problem Addressed, Contribution, Model and Technology Used, Results, Limitations, Strengths, Future Scope, Reported Top Metric (Value), Most Dominant Model, Evaluation Metrics, Hardware Setup, Monitoring Mechanism, Waste Categories, Usability, Reported Limitations, Future Improvements, and Environmental Impact. These per-study templates were then consolidated into the insight-analysis master table and the derived thematic tables presented in the Results section (Section 3.1), and figures (Figure 3, Figure 4, Figure 5, Figure 6 and Figure 7) presented in the main text.

For studies reporting multiple performance figures (e.g., across class-granularity tiers or multiple metrics), we recorded the single headline value the original authors identified as their primary result (typically overall classification accuracy, or the task-appropriate primary metric where accuracy was not reported, e.g., mAP for detection tasks or R^2^/MAPE for forecasting tasks); this is the value reported in the “Reported Top Metric” column of the insight-analysis master table (Section 3.1) and used to compute the accuracy ranges in the deep-learning performance summary (Section 3.1.1). Dataset size was recorded as the total number of images/samples used for model training and evaluation combined, as stated by the original study; where a study reported only a post-augmentation count, this was noted and used, since pre-augmentation counts were not consistently available across studies.

### 2.5. Quality Assessment

Following the selection of the primary studies, a formal quality assessment was conducted using a custom 10-item appraisal rubric developed specifically for this review. The rubric’s domains were loosely inspired by appraisal categories used in established instruments such as the NIH Quality Assessment Tool for Systematic Reviews and Meta-Analyses [32,33], but the instrument itself is a new, review-specific construction, not an adaptation, validation, or application of the NIH tool and has not been independently validated. The original NIH framework was developed for meta-analyses of comparative clinical studies and uses categorical (Yes/No/Cannot Determine) ratings culminating in an overall Good/Fair/Poor judgment. Because the studies included in this review are primarily empirical AI/ML and IoT-based computational studies rather than clinical meta-analyses, we developed the quality-appraisal framework’s core appraisal domains into a 10-item checklist with graded (0–10) scoring per item, enabling finer-grained differentiation between studies than a binary rating would allow, while retaining the original tool’s conceptual structure (research question clarity, methodological transparency, bias assessment, and reporting quality).

To ensure methodological rigor and minimize reviewer bias, the quality appraisal of all 50 included studies was carried out independently by two reviewers, who scored each study separately before reconciliation. The allocation of screening, appraisal, extraction, and coding tasks across the author team, together with the disagreement-resolution procedure, is reported in Appendix B. Each selected paper was systematically appraised using a 10-item checklist designed to evaluate internal validity and the risk of bias, applied independently by both reviewers (see Appendix B). The assessment covered critical parameters, including:

- Research Clarity: Evaluation of whether the review was based on a focused and adequately formulated research question.
- Methodological Transparency: Verification of predefined and specified eligibility criteria for study inclusion and exclusion.
- Search and Review Integrity: Assessment of the comprehensiveness of the systematic search strategy and whether titles, abstracts, and full-text articles were dually and independently reviewed.
- Comparative and Data Validity: Examination of whether each study benchmarked its approach against baseline/alternative methods, assessed dataset quality and representativeness, and reported robustness or generalizability testing (e.g., ablation studies, cross-dataset validation).
- Transparency and Data Reporting: Verification that primary data were collected, stored, and listed alongside essential study characteristics and results, and that limitations or potential sources of bias (e.g., dataset bias, overfitting risk) were explicitly discussed.

Each of the 10 criteria was scored on a 10-point scale, resulting in a maximum possible score of 100 per study. In accordance with established academic practices, a threshold score of 80 was implemented as the benchmark for inclusion. Studies achieving or exceeding this score were categorized with a “Good” quality rating, while those failing to meet this cutoff were excluded to maintain the integrity of the review. Common reasons for exclusion included incomplete methodological descriptions, a lack of formal bias assessment, or insufficient validation of reported results.

The ten criteria were selected to jointly capture four dimensions of methodological quality relevant to AI/ML- and IoT-based waste management studies: (i) research clarity and methodological transparency (Items 1–2), reflecting whether the study’s objective and methods were clearly specified; (ii) evidentiary rigor (Items 3–4, 6), covering literature grounding, comparative/baseline validation, and clarity of reported results; (iii) data and model integrity (Items 5, 8–9), covering dataset representativeness, robustness/generalizability testing, and reproducibility; and (iv) transparency and contextualization (Items 7, 10), covering explicit discussion of limitations/bias and consistency with the broader literature. Each item was scored on a 10-point graded scale rather than the binary (Yes/No) rating, allowing partial credit for studies that satisfied a criterion incompletely (e.g., a study reporting only limited robustness testing rather than none) rather than forcing a coarse binary judgment across a methodologically heterogeneous set of computational studies. A threshold of 80/100 (80%) was adopted by the author team as the inclusion benchmark, providing a reproducible, quantifiable decision rule suited to appraising a large and methodologically diverse candidate pool.

To provide a concrete illustration of the quality assessment process, Table 1 presents the evaluation of one representative study using our quality appraisal rubrics. The table outlines each of the ten criteria, the scoring outcomes, and the overall quality rating. Key criteria such as research clarity, methodological transparency, and data reporting were particularly emphasized to ensure reproducibility and rigor. This example demonstrates the systematic approach adopted across all selected studies, reinforcing the robustness and reliability of the review process. As evidenced by Table 1, the rubric provided a structured and transparent approach to evaluate each study. The consistent application of these criteria across all selected papers ensured that only high-quality and methodologically robust research was included in the review.

For descriptive context, Figure 3 additionally reports the distribution of the 50 included studies by publication year, geographical region, and journal quartile, alongside their distribution by publisher and journal venue. These journal-quartile figures describe the citation standing of the venues in which the included studies were published and are reported for descriptive/contextual purposes only; they are not used as evidence of individual study quality or risk of bias, which was instead assessed solely through the quality appraisal rubric described above (Section 2.5, Table 1). Furthermore, the distinct contribution of this SLR compared to existing literature is highlighted in the Comparative Analysis (Table 2), which benchmarks our review’s scope and methodological coverage against related review studies to position this synthesis within the existing body of intelligent waste management literature. The data extraction was distinct from the quality-assessment step described in Section 2.5: the two independent researchers (R1, R2) applied our quality appraisal rubric to rate each candidate study and determine inclusion (score ≥80/100), whereas the subsequent content extraction into the fields above was performed by the authors for all studies that passed this quality threshold.

## 3. Results

### 3.1. Overview of Findings

This subsection presents the cross-cluster evidence base and directly addresses RQ1 and RQ2 to synthesize the findings of the 50 selected studies through a thematic and interpretive lens. Within each cluster, synthesis is achieved either through aggregated pattern-level discussion across the full set of studies (as in Section 3.2.1) or, where methodological diversity within a cluster is itself a key finding (as in Section 3.2.2), through a comparative narrative structure in which each study is explicitly positioned against, and contrasted with, the preceding ones (e.g., accuracy–efficiency trade-offs, architectural choices, dataset scale) rather than described in isolation. Guided by the four-cluster taxonomy introduced earlier, the discussion progressively builds from foundational system architectures to advanced analytical intelligence and emerging paradigms. This layered organization reflects the operational pipeline of intelligent waste management systems and enables a coherent examination of technical maturity, deployment readiness, and research gaps. Table 3 summarizes the four thematic clusters, reporting for each the number of studies, the primary techniques, the main application, and the key observed limitations. A more detailed, study-level overview of the extracted insights and comparative attributes across all reviewed studies is presented in Table 4, which serves as the primary evidence base for the subsequent discussion.

Table 4 constitutes an insight analysis master table developed through systematic extraction and cross-comparison of dominant learning models, reported performance metrics, hardware and IoT configurations, monitored waste categories, usability characteristics, deployment limitations, sustainability impacts, and geographical contexts across all 50 reviewed studies, thereby enabling evidence-based synthesis and cluster-wise interpretation in the following subsections.

Regarding real-world implementation status, the hardware, monitoring, and limitation attributes extracted in Table 4 allow a broad characterization of the reviewed systems. A large share of studies, particularly in the visual recognition and predictive clusters, were developed and evaluated offline using public or curated datasets on GPU or cloud platforms, without a physical sensing or actuation component [14,16,18,39,48,68]. Thirteen studies implemented hardware prototypes based on microcontrollers or single-board computers (e.g., ESP32, Arduino, Raspberry Pi) with ultrasonic, load, or camera sensors [13,34,36,38,40,49,51,53,54,57,58,66,67], and these were generally tested in laboratory or small-scale settings. Field-level evidence is rarer: only a few studies report testing in real environments, such as river-based UAV monitoring [38] or city-level bin monitoring [41,67], and none of the reviewed studies report multi-site or long-term operational deployment. Overall, the evidence indicates that most reviewed systems remain at the prototype or pilot stage rather than routine societal use. We note, however, that this assessment reflects what the included studies report; commercial or municipal deployments that are not documented in peer-reviewed empirical articles fall outside the scope of this review.

Collectively, this section is structured to directly address the formulated research questions by examining both comparative technical performance (RQ1) and real-world deployment readiness (RQ2) across the reviewed intelligent waste management systems.

IoT-enabled smart bin architectures form the physical and sensing backbone of intelligent waste management systems [13,41]. Across the reviewed literature, these systems consistently emphasize real-time monitoring of bin fill levels, environmental conditions, and collection status through sensor-driven infrastructures [50]. Representative implementations commonly integrate ultrasonic, weight, and gas sensors with low-power microcontrollers and wireless communication technologies such as LoRa, GSM, and Wi-Fi to enable continuous data transmission and remote supervision [53]. The generalized operational workflow shared across these studies ranges from data acquisition and wireless transmission to cloud-based processing and actuation [58,67].

Across the IoT-focused studies, a clear majority report deployments restricted to pilot-scale or semi-controlled environments, underscoring the early-stage maturity of hardware-centric intelligent waste infrastructures [36,40,49,51,58].

Despite architectural variations, a common operational pattern emerges across IoT-enabled waste systems, consisting of sensing, wireless data transmission, cloud-based storage, and rule-based alert generation [13,36,41,53,58]. While this pipeline has been shown to improve collection efficiency and reduce overflow incidents, the intelligence embedded in most implementations remains largely reactive rather than adaptive [54]. Decision-making is typically threshold-driven, with limited incorporation of predictive or learning-based mechanisms, thereby constraining system responsiveness under dynamic urban conditions [49]. A majority of studies report pilot-scale deployments restricted to controlled environments such as university campuses or small municipalities, revealing a persistent gap between prototype validation and city-wide scalability [40,49,51,58,66]. These limitations are systematically captured in Table 3 under the deployment scale and system usability dimensions.

Importantly, this hardware-centric focus exposes a structural dependency on higher-level intelligence. As a result, IoT architectures alone are insufficient to address waste stream heterogeneity or classification complexity. This limitation naturally motivates the transition toward vision-based intelligence, discussed in the following subsection.

Building upon the sensing infrastructure provided by IoT systems, deep learning-based visual recognition has emerged as the dominant approach for automated waste classification in intelligent waste management pipelines [17,34]. The majority of representative studies in this cluster employ convolutional neural networks (CNNs) and object detection architectures to categorize waste into recyclable, organic, hazardous, and residual classes [14,44]. A comparative mapping of the dominant deep learning architectures and their reported performance ranges across the reviewed studies is summarized in Table 5. A comprehensive statistical summary is shown in Figure 4, revealing that while CNNs and YOLO variants represent the most frequently utilized architectures, Hybrid models maintain the most consistent high-accuracy profile across the reviewed literature.

More than half of the reviewed visual recognition studies report classification accuracies exceeding 90%, primarily under controlled experimental conditions and curated datasets. A clear trend is the widespread adoption of transfer learning using architectures such as ResNet, EfficientNet, MobileNet, and YOLO variants. These models consistently report high classification accuracies, particularly when trained on well-curated datasets under controlled lighting and background conditions.

However, cross-study comparison suggests that reported performance may be shaped as much by dataset visual uniformity and curation quality as by architectural novelty alone, though the heterogeneity of reporting practices across studies limits any firm attribution of this pattern. Figure 5 presents dataset size against reported accuracy across the accuracy-reporting subset of studies; no clear increasing trend is visually apparent, indicating that a larger dataset alone does not consistently correspond to higher reported accuracy in this literature. This should be read as a descriptive observation rather than a controlled test of the effect of dataset size, since the underlying studies also vary in class count, task difficulty, augmentation strategy, and architecture, any of which could independently influence reported performance.

A related caveat applies to hybrid and optimizer-assisted models, which record some of the highest headline values in this review. Several included studies combine a base learner with a metaheuristic or search-based optimizer, for example VGG16 with a Cat Swarm Optimized Random Forest [42], a Decision Tree with Binary PSO [43], an improved ResNet with an artificial ecosystem-based optimizer [50], a kernel soft extreme learning machine with dynamic bald eagle search optimization [58], and a dual-ensemble CNN with AMIS optimization [59]. In such studies, the reported accuracy reflects the base learner, the optimizer, feature selection, and hyperparameter tuning jointly, and the studies differ in optimizer, search budget, and tuning protocol, which this review did not extract. The high accuracies reported for hybrid systems should therefore not be read as evidence of architectural superiority alone: they may be confounded by optimizer choice, hyperparameter tuning, and data curation. Because component-level coding (base learner versus optimizer) was not part of our extraction template, this review cannot attribute these gains to any single component and does not rank optimizers against one another.

Several works explicitly acknowledge performance degradation under real-world conditions involving occlusion, contamination, or mixed waste scenarios [16,18,47,68], a limitation also reflected in the dataset diversity attributes of Table 3. Another notable observation is the limited integration between visual classifiers and upstream IoT sensing layers. Most vision-based systems operate as standalone modules evaluated offline, with minimal consideration for real-time constraints, edge deployment, or hardware-resource limitations. This disconnect restricts practical adoption and underscores the need for tighter coupling between perception and system-level decision-making. Consequently, attention has shifted toward predictive analytics to bridge perception and operational planning, as discussed next.

Predictive analytics represents a critical transition from perception-driven intelligence toward proactive and data-informed waste management strategies [35,43]. Representative studies within this cluster apply machine learning models, including regression techniques, random forests, gradient boosting, and recurrent neural networks, to forecast waste generation patterns, bin fill levels, and collection demand [37,63]. The alignment between predictive objectives, learning models, and their corresponding operational outcomes is systematically detailed in Table 6 [22,65].

Only a limited subset of predictive studies validate model performance across multiple cities or extended temporal horizons, indicating restricted generalizability in real-world operational contexts.

The findings indicate that predictive models significantly improve operational efficiency when historical and temporal data are available. However, most implementations remain context-specific, trained on data from single cities or limited time horizons. This restricts generalizability and raises concerns regarding robustness under seasonal, demographic, or behavioral shifts [37,63,70]. Furthermore, predictive modules are often evaluated independently of classification accuracy, resulting in fragmented system intelligence, a pattern observable across clusters in Table 7.

The absence of unified pipelines integrating sensing, classification, and prediction highlights a structural gap in current research. This gap has catalyzed interest in domain-specific solutions and emerging technologies aimed at achieving end-to-end intelligence, which are examined in the final cluster.

The final cluster encompasses specialized waste streams and emerging technological paradigms that extend beyond conventional municipal solid waste management practices [15,52]. These studies primarily address e-waste, medical waste, construction debris, and hazardous materials, often operating under stricter regulatory, safety, and handling constraints compared to general municipal waste systems [56,60].

Emerging technologies such as edge AI, federated learning, blockchain-based traceability, and UAV-assisted monitoring are increasingly proposed as solutions to scalability, privacy, and data heterogeneity challenges. The distribution of these technologies across the reviewed studies and their intended system-level roles are illustrated in Figure 6. While these approaches demonstrate conceptual promise, most remain at the simulation or framework-design stage, with limited empirical validation [59,62].

From an environmental perspective, the reviewed systems demonstrate indirect sustainability benefits through reduced overflow events, optimized collection routes, and improved recycling accuracy; however, only a small number of studies quantitatively evaluate emissions reduction, energy consumption, or lifecycle impacts, representing a critical gap in the current literature.

#### 3.1.1. Synthesis for RQ1: Reported Technical Performance

Across the 50 studies, 37 reported an accuracy-type headline metric for classification or detection. Of these, approximately 32 (about 86%) reported values of 90% or higher, whereas five reported lower values [42,44,55,56,69]. Regression and forecasting studies (n=6) reported $R^{2}$ values between 0.88 and 0.999, and correlation coefficients up to 0.996 [22,35,37,61,62,65]. By model family (Table 5), reported accuracy ranged from 85.0 to 98.5% for YOLO variants to 96.0–99.9% for hybrid/ensemble models, with 35 of the 37 accuracy-reporting studies included in Table 5. The two remaining studies also report an accuracy value but were excluded from Table 5 and Figure 5 because they do not use one of the listed image-classification model families: Ref. [56] applies a zero-shot/few-shot vision-language model (CLIP), and [20] reports forecasting accuracy for an LSTM pollutant-prediction model. Figure 5 plots classification accuracy against dataset size for the same 35 studies underlying Table 5; no consistent pattern is visually apparent between dataset size and reported accuracy across this subset, though this descriptive observation does not control for confounding factors such as class count, task difficulty, augmentation strategy, or architecture, and should not be read as evidence that dataset size has no effect on performance. Most studies used medium-sized datasets (29 medium, 16 large, and 5 small; Figure 4). Only 3 of the 50 studies compared their approach with a traditional, non-AI baseline; therefore, claims of superiority over conventional practice cannot be systematically supported.

#### 3.1.2. Synthesis for RQ2: Scalability and Deployment Readiness

Of the 50 studies, 13 implemented hardware prototypes on microcontrollers or single-board computers, and none reported multi-site or long-term operational deployment. Field-level evidence was limited to a few studies [38,41,67]. Regarding the input data underpinning readiness (RQ2), 36 of the 50 studies rely primarily on RGB imagery, five of which also pair the camera with ultrasonic, load or proximity sensors but process the two streams in separate pipelines [13,36,41,49,66]. Six studies use IoT sensor streams alone [20,21,43,53,54,58], and eight use tabular socioeconomic, geospatial or chemical-composition variables [19,22,35,37,61,62,65,70]. Image-based systems therefore depend on curated visual datasets and GPU training, whereas sensor- and tabular-based systems depend on site-specific historical records or laboratory measurements, which limits their transferability. The gap frequencies in Figure 7 show that limited dataset diversity (24 studies), lack of real-world validation (13), and classification gaps (11) were the most common barriers, followed by integration gaps (9), model complexity (8), federated/edge AI (6), interoperability (5), and explainability/fairness (4). Deployment maturity therefore remained low across clusters (Table 7).

For municipal stakeholders and policymakers, these findings suggest that future investments should prioritize integrated, end-to-end intelligent waste management platforms rather than isolated technology deployments. Nevertheless, the interpretation of these findings should be considered in light of the methodological, data availability, and deployment-scale limitations discussed in the subsequent section.

### 3.2. Cluster-Wise Discussion

In this section, we briefly discuss the core perspectives presented by the reviewed studies and synthesize their findings according to the four clusters defined earlier. We interpret the evidence cluster by cluster, comparing individual studies, design trade-offs, and limitations. A total of 50 peer-reviewed articles were systematically analyzed to understand how IoT, machine learning, deep learning, and data-driven optimization techniques are being applied to intelligent waste management systems.

Rather than discussing each study in isolation, this section groups the literature based on architectural focus, modeling strategy, operational intelligence, and emerging application domains. The purpose is to highlight recurring design patterns, dominant technologies, methodological trends, and shared limitations observed across the reviewed works. Each subsection below summarizes the key insights reported by the studies within that cluster, allowing readers to clearly understand the technological evolution, practical challenges, and research directions shaping smart waste management systems. Cluster assignment followed an inductive, qualitative thematic synthesis approach rather than a quantitative or algorithmic clustering method. Each included study was assigned to the single cluster that matched its primary technological focus and stated contribution, i.e., IoT hardware/ sensing infrastructure (Cluster 1), deep learning-based visual classification (Cluster 2), predictive/optimization analytics (Cluster 3), or domain-specific/emerging applications (Cluster 4). For studies spanning more than one theme, assignment was based on the primary contribution emphasized by the study’s authors (e.g., a paper combining CNN classification with IoT sensing was assigned to Cluster 2 if its central contribution was the classification model, or Cluster 1 if its central contribution was the sensing/hardware system).

#### 3.2.1. Cluster 1: IoT-Enabled Smart Bin Architectures: Foundations of Intelligent Waste Systems

This cluster comprises fourteen studies focused on IoT-based hardware architectures for smart waste monitoring and automated bin management [13,20,36,40,41,49,50,51,53,54,58,61,66,67]. Rather than centering algorithmic novelty, these works emphasize sensor integration, embedded systems, cloud connectivity, and real-time monitoring infrastructure, transforming conventional bins into network-connected systems.

##### Sensors and Data Modalities

Ultrasonic distance sensing for fill-level detection is the near-universal baseline across the cluster [13,36,41,53,58,66], frequently paired with load/weight sensors [13,41,53] and, in a subset of studies, an onboard camera for visual classification [36,40,41,49,50,66,67]. A smaller number of studies extend sensing beyond fill-level and image data toward environmental parameters, including odor/air-quality sensing for pollutant forecasting [20] and organic-waste chemical-property monitoring for C/N-ratio prediction [61]. Beyond ultrasonic, load, gas, and camera sensing, passive RFID offers a further low-cost identification and monitoring modality for smart infrastructures. Outside the waste domain, commodity RFID tag arrays have been used for material classification and humidity sensing with accuracy above 96% across four indoor environments [74]. RFID-based sensing is not among the sensor modalities extracted from the reviewed studies.

##### Learning and Fusion Methods

Where a classification component is present, CNN backbones dominate: ResNet variants [13,41,49,50], DenseNet [36], VGG16 [51], and MobileNetV2 [67], typically deployed as a standalone module rather than fused with the sensor stream at a feature level. Optimization-driven approaches appear where the primary task is prediction or routing rather than image classification: a genetic algorithm for route optimization [53], dynamic bald eagle search optimization with a kernel soft extreme learning machine for bin-status prediction [58], GAN-based generation combined with ARIMA forecasting for e-waste sorting [54], and an LSTM for pollutant-level forecasting [20]. Across the cluster, sensor data and vision data are largely processed through separate pipelines rather than a unified multimodal fusion architecture, a gap noted explicitly in Section 4.

##### Validation Setting

Most studies report validation on a single prototype or simulated environment, e.g., simulation-only validation in a Wokwi environment [36], and single-site/single-city prototype testing across the remainder of the cluster [13,40,49,51,53,58,66]. City-scale field deployment claims are made in two studies [41,67], though neither reports multi-site replication.

##### Quantitative Evidence

Reported classification accuracies cluster tightly in the 90-99% range [36] (94.63%), [13] (95.31%), [41] (95%), [49] (94.44%), [50] (∼99%), [53] (96.1%), [58] (96.42%), [67] (96.57%), while the pollutant-forecasting and C/N-ratio studies report different metric families entirely (90% forecasting accuracy [20]; $R^{2}=0.97$ on the test set for C/N-ratio regression [61]), consistent with the broader caution in Section 4 against pooling these into a single comparable figure.

##### Deployment Maturity

On the deployment-readiness axes defined in RQ2 (Section 2), this cluster sits exclusively at the laboratory-prototype or single-site pilot stage; only [41,67] report anything approaching multi-station or city-scale operation, and none report multi-site replication, extended operating-duration data, or systematic communication-reliability testing under field conditions. Cross-domain IoT work illustrates which sensing-layer quantities an operational assessment should record separately from classifier accuracy. In the RFID-based RFbook system [74], the authors report frequency-hopping latency against accuracy (12 channels, 1–2 s per classification), a fall in accuracy from about 95% to 80% when object placement differed between training and testing, and a rise in false-negative rate from 2.7% to 5.2% across repeated sessions without model adaptation. Such hardware-, communication-, and drift-level measurements are largely absent from the smart-bin studies in this cluster. Paper [74] is a laboratory prototype in a non-waste domain; it is cited only as a methodological reference for the RQ2 readiness criteria, is not one of the 50 included studies, and does not alter any count in this review.

##### Limitations and Evidence Gaps

The cluster collectively shows persistent gaps in communication latency (e.g., platform-level update delays [36]), narrow waste-category coverage (as few as two to six classes across most studies), limited dataset diversity, and an almost complete absence of systematic real-world robustness testing (weather, occlusion, network failure). These limitations indicate that hardware-centric IoT architectures provide the operational backbone of smart waste management systems, but that their effectiveness increasingly depends on the intelligence and robustness of the downstream data-interpretation layer, motivating the next cluster’s focus on deep learning-driven visual recognition (Section 3.2.2).

#### 3.2.2. Cluster 2: Deep Learning for Visual Waste Recognition: Advancing Automated Classification

This cluster shifts the analytical focus from IoT-centric hardware systems (Section 3.2.1) to deep learning–driven visual intelligence for automated waste classification. It synthesizes insights from twenty studies that examine how CNN-based architectures and their optimized variants influence classification performance. The reviewed works emphasize key factors such as model design, dataset scale, feature representation, computational efficiency, and real-time feasibility. Unlike IoT-focused research that prioritizes sensing and system integration, these studies concentrate on the algorithmic core of visual perception, addressing challenges including class imbalance, visual ambiguity, limited training data, and deployment constraints. Collectively, the findings demonstrate a methodological progression from small-scale CNN classifiers to large curated datasets, lightweight architectures, and real-time detection models, reflecting the growing maturity of deep learning approaches in practical waste classification.

A foundational contribution within this cluster is provided by Nahiduzzaman et al. [34], who address the limitation of fragmented datasets by introducing the TriCascade WasteImage dataset (35,264 images). Their proposed DP-CNN, combined with an ensemble ELM classifier, demonstrates that dataset scalability directly enhances robustness while maintaining the low computational complexity required for embedded systems. The framework achieved high performance across three tiers: 96% accuracy (binary), 91% (nine-class), and 85.25% (thirty-six-class). However, despite these gains, the authors acknowledge residual overfitting risks due to multi-source heterogeneity and class imbalance, suggesting that volume alone does not fully resolve generalization challenges.

Shifting from the data-centric focus of [34], Shetty et al. [14] prioritize architectural diversity to optimize performance under data constraints. By proposing lightweight models EcoMobileNet and EcoDenseNet, they achieve a high accuracy of 98.29% without expanding the dataset size. However, their reliance on data augmentation and ensemble learning increases computational overhead, creating a trade-off between accuracy and real-time feasibility. While [14] demonstrates that architectural refinement can compensate for limited data, it results in higher inference complexity compared to the low-complexity framework in [34].

While [14] emphasizes architectural efficiency, Ahmad et al. [39] pivot back to dataset scale, utilizing 15,535 images across 12 waste categories. Their results confirm that deep ResNet-based models outperform classical architectures, reinforcing CNN dominance in multi-class recognition. However, unlike the lightweight designs in [14], increased model depth entails high computational costs that hinder edge deployment. This underscores a core trade-off within this cluster: the pursuit of accuracy through deeper architectures frequently conflicts with real-time resource constraints.

To bridge the gap between accuracy and efficiency, Madhavi et al. [16] introduce SwinConvNeXt, a hybrid architecture combining CNN-based local feature extraction with transformer-inspired global context modeling. Unlike pure transformer approaches, this CNN-centric design preserves efficiency while enhancing contextual awareness. Compared to the resource-heavy models in [39], SwinConvNeXt achieves near-perfect accuracy (98.97%) with significantly lower computational overhead, specifically targeting real-time deployment. This work represents a pivotal shift toward hybrid designs that successfully mitigate the accuracy–efficiency trade-off identified in earlier studies.

Transitioning from classification to real-time object detection, Rajeev et al. [17] evaluate YOLOv5, YOLOv7, and YOLOv8 for e-waste recognition. These models prioritize inference speed for robotic sorting, with YOLOv8 demonstrating superior performance (mAP@0.5 of 0.82; precision of 0.83). However, the study’s small dataset reintroduces the overfitting concerns first raised in [34], reinforcing that even advanced detection frameworks remain dependent on the large-scale data diversity highlighted early in this cluster.

Moving away from end-to-end deep learning, Gaurav et al. [42] evaluate a hybrid pipeline using VGG16 for feature extraction followed by a swarm-optimized Random Forest classifier. While this approach reduces computational burden aligning with real-time IoT deployment goals, its lower accuracy (85%) suggests that decoupling feature extraction from classification may compromise representational power. This contrast reinforces the advantages of the end-to-end learning models seen in [16,17], where integrated feature learning leads to superior performance.

Li et al. [18] explicitly target the efficiency concerns of this cluster by optimizing a ResNet architecture with depthwise-separable convolutions and redundancy-weighted feature fusion. This design directly addresses the computational bottlenecks of deep CNNs identified in [39] while retaining their accuracy benefits. Although evaluated on the smaller TrashNet dataset, the model achieves competitive performance with fewer parameters and faster inference. This study reinforces the cluster-wide trend toward lightweight, real-time-capable CNN optimization as a standard for modern waste classification.

A significant advance in this direction is presented by Sayem et al. [44], addressing category diversity and industrial robustness through the WaRP dataset (28 categories) and a dual-stream DenseNet-201 + MaxViT architecture. Their work demonstrates that hybrid feature fusion significantly improves performance in complex recycling streams, with benchmarking showing that GELAN-E outperforms YOLOv5 and YOLOv8 under challenging lighting and occlusions. However, the moderate classification accuracy (83.11%) and high computational overhead highlight a persistent gap: while CNN–Transformer hybrids scale well for complex datasets, they still face significant hurdles for real-time edge deployment compared to the optimized models in [16,18].

Complementing the visual-centric studies, Ghorbani et al. [19] pivot to data-driven waste generation prediction within the construction sector. Utilizing structured project data rather than images, the study demonstrates that XGBoost consistently outperforms alternative ML models, achieving over 98% precision across project phases. While methodologically distinct from the CNN-based approaches in [39], this work reinforces a core insight of the cluster: model selection must strictly align with data modality and operational goals. Furthermore, the integration of SHAP-based explainability introduces a layer of transparency that addresses the “black box” nature of earlier models, foreshadowing a shift toward interpretable systems in waste management.

Returning to image-based recognition, Chhabra et al. [45] focus on architectural refinement within conventional CNNs, proposing a multi-layer model utilizing LeakyReLU activation and dropout regularization for binary household waste classification. Achieving 93.28% accuracy, the study demonstrates that optimized activation and regularization can effectively mitigate overfitting and class imbalance. However, compared to the industrial-scale multi-class frameworks in [44], this work remains limited to binary classification and GPU-dependent training. This contrast underscores the persistent gap between high-performing laboratory-validated models and the scalable, resource-efficient systems required for real-world deployment.

Directly addressing scalability, Chacón-Albero et al. [47] propose a lightweight, quantized framework tailored for IoT and edge deployment. By balancing a 27,396-image dataset across seven categories and applying 8-bit post-training quantization, the study achieves 92.2% accuracy with a quantized YOLOv11n-cls model while drastically reducing computational costs. In contrast to the heavy dual-stream models in [34] and the GPU-centric design in [45], this work prioritizes efficient inference on resource-constrained hardware. Additionally, the introduction of ensemble aggregation ensures that this reduction in model size does not compromise the robustness required for real-world recycling environments.

While [47] prioritizes efficiency, Celik [48] explores the opposite extreme by maximizing performance through multi-layer feature fusion. By integrating EfficientNetB0, InceptionV3, HyperColumn fusion, and XGBoost, the proposed model achieves near-perfect accuracy (up to 99.87%). While this result underscores the efficacy of multi-resolution feature learning, it reinforces the recurring trade-off in this cluster: reported gains in accuracy often coincide with higher computational and energy costs. This disparity highlights a significant barrier for mobile or IoT applications, which remain inaccessible to such high-fidelity models without further optimization.

Bridging the gap between performance and efficiency, Malik et al. [55] demonstrate that lightweight architectures specifically EfficientNet-B0—can achieve competitive accuracy (85%) with a fourfold reduction in FLOPs compared to larger models. By utilizing region-specific fine-tuning, the model effectively adapts to localized waste patterns, offering a scalable solution for urban environments in developing regions. Compared to the high-resource ensemble in [48], this work highlights that strategic fine-tuning of lightweight models provides a practical compromise; however, the lack of real-time testing or IoT integration remains a hurdle for immediate field deployment.

The cluster culminates with Naznine et al. [57], who explicitly address the unresolved challenges of computational efficiency, interpretability, and real-world validation. Their proposed PLDs-CNN with Ridge-ELM achieves state-of-the-art accuracy (99%) with a model footprint of only 12.7 MB and millisecond-level inference, validated on NVIDIA Jetson edge devices. Crucially, the integration of SHAP-based explainability provides real-time transparency into model decisions, answering the “black-box” concerns raised implicitly in earlier works. This study is among the few in the cluster to report high accuracy, low latency, and explainability together, suggesting these properties are not mutually exclusive in practical waste classification systems, though this remains a single-study result rather than a demonstrated general pattern.

Following the emergence of interpretable and edge-deployable models in Cluster 2, Ahmad et al. [64] extend the efficiency-focused efforts of the cluster by proposing a custom CNN that reduces parameter count from nearly 10 million to approximately 0.5 million. By maintaining 92.5% accuracy through a global average pooling (GAP)-based design, the study demonstrates that high performance is possible without resource-intensive backbones like VGG16 or ResNet. This work provides a practical blueprint for low-power device deployment in developing-country contexts. However, the restriction to only four waste categories highlights a persistent trade-off: while architectural simplification maximizes efficiency, it may limit the model’s generality compared to more complex multi-class systems.

While [64] prioritizes custom lightweight designs, Ahmed et. al. [68] leverage transfer learning with pre-trained models to maximize accuracy. Systematically comparing CNN, DenseNet169, MobileNetV2, and ResNet50V2 on a Middle Eastern–centric dataset, the study finds that ResNet50V2 achieves a superior 98.95% accuracy. In contrast to the parameter-efficient philosophy of [64], this work reinforces the value of deep representational capacity when computational resources are available. Together, [64,68] illustrate the central tension in this cluster: accuracy-oriented transfer learning versus efficiency-oriented custom design, both essential depending on specific deployment constraints.

Expanding beyond centralized benchmarking, Khan et al. [69] evaluate ten CNN architectures on the large-scale TrashBox dataset within a federated learning framework. This study addresses critical gaps in data privacy and cross-site scalability that centralized training paradigms like those in [64,68] cannot resolve. While identifying ResNeXt-101 as the performance leader and ShuffleNetV2 as a lightweight alternative, the authors introduce a new system-level dimension to the cluster: collaborative learning. By allowing models to train across distributed facilities without sharing raw data, this work shifts the perspective from static deployment to a dynamic, privacy-preserving ecosystem for smart city waste management.

Moving beyond supervised paradigms, Al Duhayyim et al. [71] introduce deep reinforcement learning (DRL) for waste classification. Their IDRL-RWODC framework combines Mask R-CNN for detection with a Deep Q-Learning Network (DQN) for classification, achieving 99.3% reported accuracy. This approach extends previous CNN and hybrid models by enabling adaptive decision-making rather than static classification. However, the high computational demands of DRL and the reliance on limited datasets echo persistent challenges regarding real-time deployment and generalization, suggesting that while DRL improves accuracy, its operational cost remains a significant barrier.

Adaptability is further emphasized by Bhattacharya et al. [72] shift the focus toward lifelong learning by proposing a self-adaptive system that evolves through user feedback. Unlike the static pipelines prevalent in Cluster 2, this framework retrains itself when misclassifications occur to accommodate changing waste compositions. Although a significant gap between training (98%) and validation (80%) accuracy persists, the study introduces a vital conceptual shift toward system adaptability. This approach complements the federated vision of [69] and the reinforcement-based adaptability of [71], moving the field closer to autonomous, self-correcting waste management ecosystems.

Finally, Kumar et al. [73] provide a foundational baseline for Cluster 2 by evaluating the accuracy–speed trade-off in real-time object detection using YOLOv3 and YOLOv3-tiny. The high mAP (94.99%) achieved by YOLOv3 validates the feasibility of automated waste detection, while the performance degradation in the “tiny” variant foreshadows the urgent need for the lightweight optimizations later seen in [47,57,64]. This work anchors the cluster’s methodological evolution, establishing the original performance benchmarks that modern hybrid and federated systems continue to reference.

The YOLO-based studies in this cluster all operate on RGB imagery. Sensor-coupled recognition represents a distinct design. In the neighbouring domain of mining waste, Wang et al. [75] couple a purpose-built differential-electrode electrical capacitance tomography (ECT) sensor with YOLO-series detectors (YOLOv8n–YOLOv12n), reporting recognition accuracies of 93.2–96.7% on ECT images of coal gangue. This study lies outside the review’s municipal scope and eligibility window and is cited only to illustrate that modern YOLO architectures can be paired with non-optical sensing rather than relying solely on standard vision inputs; its results are not comparable with the RGB-based accuracies in Table 5 and are not included in the review’s counts.

The evolution of Section 3.2.2 marks a transition from basic CNN classifiers to sophisticated, edge-optimized, and interpretable architectures. While these advancements have mastered the perception layer, achieving high accuracy and real-time feasibility, the reviewed literature remains largely perception-centric. Most studies treat visual recognition as the final objective rather than an enabling mechanism for broader operational logic.

This limitation suggests that the true value of advanced perception lies in its integration into higher-level decision frameworks. In practical ecosystems, classification must serve as the foundation for forecasting generation, optimizing collection routes, and allocating resources. Consequently, the research trajectory shifts from “what is being detected” to “how should the system respond.” This motivates Section 3.2.3, which moves beyond image-based recognition toward data-driven predictive analytics and operational optimization frameworks.

#### 3.2.3. Cluster 3: Predictive Analytics and Operational Optimization: From Perception to Decision-Making

Unlike Section 3.2.2, which primarily focused on improving visual recognition accuracy, Section 3.2.3 shifts attention toward higher-level intelligence, specifically forecasting, optimization, routing, resource allocation, and policy-level strategic planning. The nine studies in this cluster extend waste management research beyond classification to proactive, data-driven decision-making frameworks.

Collectively, these works demonstrate how machine learning and deep learning models can transform waste management systems from reactive service models into predictive and optimized operational ecosystems. The reviewed studies incorporate time-series forecasting, regression modeling, routing optimization, energy estimation, remote sensing analytics, and economic-environmental impact analysis to improve efficiency, reduce cost, and lower greenhouse gas emissions. Predictive and optimization studies address system-level problems such as routing, network planning, and infrastructure connectivity [76], so they are judged by operational objectives (e.g., forecast error, cost, and emissions reduction) rather than by the accuracy-based metrics used for image classification in Cluster 2.

To systematically synthesize the methodological diversity and operational insights of this cluster, the findings are summarized in two structured Table 8 and Table 9.

Although Section 3.2.3 establishes robust forecasting and optimization mechanisms for municipal operations, most studies remain focused on generalized MSW systems at the city or policy level. Emerging research, however, increasingly addresses domain-specific waste streams such as e-waste, plastic waste, hazardous waste, construction debris, organic composting systems, and circular economy applications. These works extend beyond operational efficiency to explore sustainability innovation, specialized recycling technologies, environmental monitoring, and emerging smart-city frontiers.

This progression motivates the next Section 3.2.4, which shifts the focus from generalized predictive and optimization frameworks to domain-specific applications and emerging research frontiers that redefine how intelligent waste management systems are deployed across specialized sectors and sustainability ecosystems.

#### 3.2.4. Cluster 4: Domain-Specific Applications and Emerging Frontiers

Recent research in waste management has shifted toward domain-specific and emerging frontier applications, where traditional municipal-level AI models are insufficient to address specialized waste streams. Section 3.2.4 synthesizes studies focusing on e-waste, medical waste, disaster debris, plastic pollution, hazardous waste, and illegal dumping, highlighting innovative AI technologies, deployment readiness, and sustainability contributions.

Table 10 summarizes the key studies, technological approaches, performance metrics, deployment maturity, and environmental impact for domain-specific waste management systems. The table illustrates how hybrid deep learning architectures, UAV-integrated edge AI, lightweight CNNs, and vision-language models are applied to highly specialized waste domains. The reviewed studies demonstrate that AI can enhance operational efficiency, enable real-time monitoring, and reduce human exposure to hazardous materials, while also providing guidance for policy-level interventions and sustainable infrastructure development.

Table 11 complements this by presenting emerging technological frontiers and their potential ecosystem integration, emphasizing scalability and research gaps. This helps highlight the next-generation landscape for intelligent waste management, where AI, IoT, edge computing, and hybrid approaches converge.

Collectively, the studies in Section 3.2.4 illustrate the diverse technological spectrum of domain-specific waste management systems, ranging from edge-AI-enabled UAV monitoring to lightweight, WHO-compliant medical waste classifiers and policy-driven predictive models for hazardous waste. The cluster underscores that deployment maturity varies significantly across domains, with medical and e-waste systems approaching pilot-level operationalization, whereas VLM-based and disaster-focused models remain emerging but highly promising. On the other hand, AI perception and physical resource recovery are distinct stages of the e-waste chain. Image-based classification identifies and sorts waste objects; it does not recover material. Downstream recovery is a separate metallurgical problem, for example capturing, silver from waste silicon photovoltaic cells using a tin–bismuth melt [77]. Such work belongs to the circular-economy chain but is not evidence of classification accuracy or intelligent sensing performance.

These insights naturally motivate a critical analysis of the field, focusing on data limitations, real-world applicability, scalability, environmental impact, and cross-domain generalizability. The next section synthesizes these observations to identify overarching trends, gaps, and directions for future research.

## 4. Discussion

The review of fifty studies on waste classification and intelligent management systems reveals persistent cross-cutting limitations that constrain real-world scalability, robustness, and sustainability of AI- and IoT-driven solutions. Despite advances in sensing, perception, prediction, and domain-specific applications, most reported gains remain confined to controlled settings, exposing a gap between laboratory performance and operational deployment.

A dominant limitation is dataset scarcity and bias. Visual classification and detection studies often rely on small or moderately sized image datasets collected under controlled lighting, background, and camera conditions, limiting generalization to mixed, cluttered urban environments [14,16,18,34,39,47,48,64,68]. Domain-specific datasets e-waste, disaster, and medical waste are smaller, curated, and biased, underrepresenting rare or ambiguous waste types [14,15,52,60]. While augmentation and synthetic generation are common, they rarely capture true real-world variability [15,44,59].

Waste categorization is often narrow. Many studies adopt binary or coarse-grained classes (recyclable vs. non-recyclable), oversimplifying municipal, industrial, and hazardous waste complexity [13,42,45,52,55]. Multi-class approaches frequently exclude hazardous, wet, or degraded waste, limiting safety and applicability [40,59,62]. Fine-grained or domain-specific classification exists but remains fragmented and lacks unified benchmarks [56,60].

Methodologically, high-performing CNN, ensemble, and CNN–Transformer models impose substantial computational costs, hindering real-time deployment on edge devices like smart bins, UAVs, or mobile robots [16,44,48,59]. Lightweight models improve efficiency but often sacrifice accuracy and are sensitive to class imbalance [47,57,64,66]. Systematic evaluation of energy use, latency, and reliability is limited [38,60,69].

Hybrid and optimizer-assisted systems add a further confound: because their reported accuracy reflects the base learner, optimizer choice, hyperparameter tuning, and dataset curation jointly, their high headline values cannot be attributed to architectural superiority alone, and optimizer-to-optimizer rankings cannot be reliably drawn from the available literature (RQ1). Separating these components, and recording the validation protocol used in each study, is a priority for future reviews.

Integration gaps persist. Classification outputs are rarely linked to route optimization, scheduling, or policy support [18,34,39,47], while predictive and optimization studies often lack real-time perception integration [35,37,43,46,63]. IoT-enabled deployments remain largely pilot-scale, with robustness under weather, occlusion, and communication failures seldom assessed [36,38,40,51,52,58,59,67].

Interpretability and fairness are underexplored, and the explanation methods reported across studies answer conceptually different questions rather than constituting a single “interpretability” category. We distinguish four types: (i) visual/spatial attribution (Grad-CAM), which highlights image regions driving a CNN’s decision, used in [14,34]; (ii) instance-level feature attribution (SHAP), which quantifies each input feature’s contribution to an individual prediction, used in [19,57]; (iii) global feature-importance ranking, which identifies which features are most predictive on average across a dataset (e.g., ash content as the dominant predictor via feature-importance analysis in [61]); and (iv) none of the reviewed studies in this set report a formal input-perturbation sensitivity analysis in the strict robustness-testing sense, though several vision-based studies report qualitative robustness observations (e.g., performance degradation under occlusion or mixed waste) without a systematic sensitivity protocol [16,18,47,68]. Across the reviewed literature, these methods are applied narrowly, typically to a single model or dataset and are rarely accompanied by dedicated subgroup or bias evaluation, so their use should be read as a diagnostic aid for the specific model reported rather than a general fairness or trust guarantee. Model decisions in the remaining studies are not accompanied by any reported explanation method, limiting trust and adoption; this includes the forecasting and regression studies [21,65], cited here as examples.

Region-specific training raises transferability concerns, while federated and distributed learning remain underutilized [52,55,62,68,69].

Domain-specific solutions (e-waste, medical, disaster, hazardous) frequently evolve in isolation, reducing interoperability and systemic impact [15,59,60,62].

**Figure 8:**
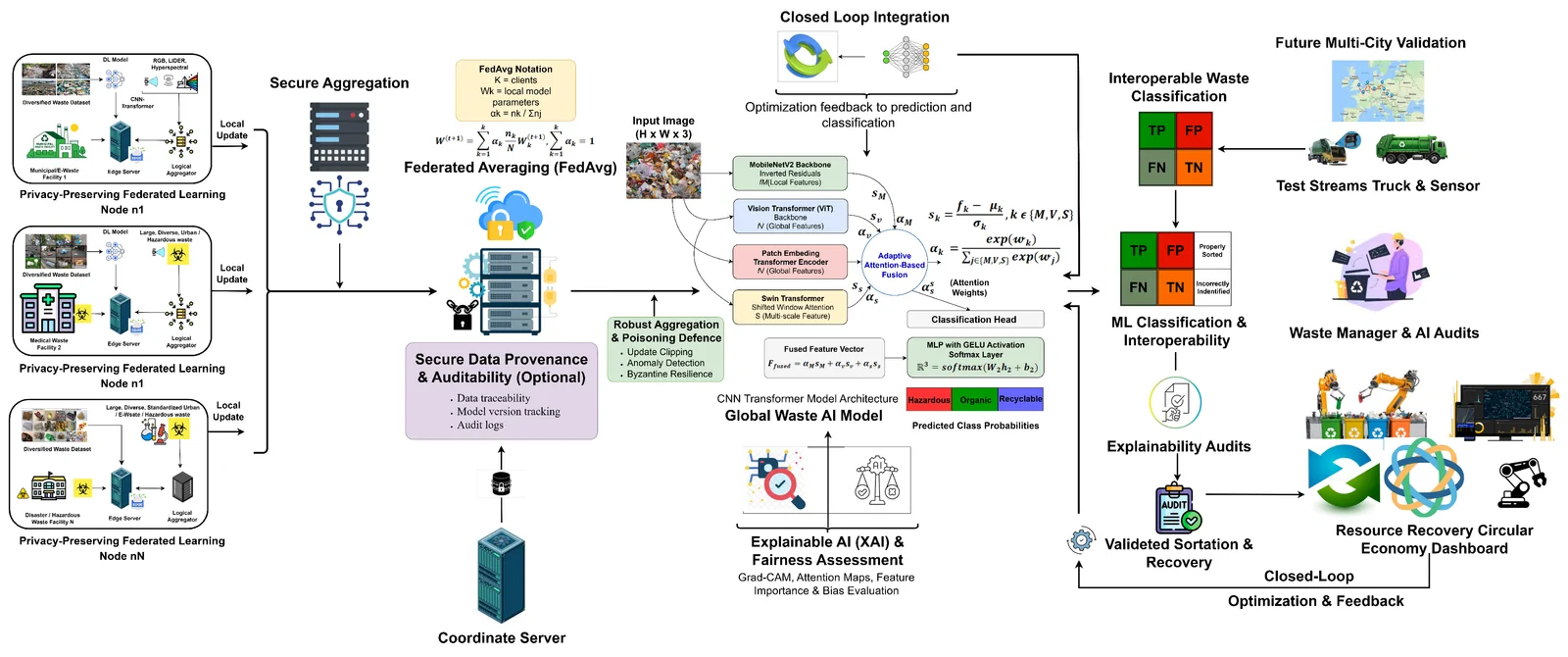
Illustrative research agenda (not a systematically derived architecture). This schematic proposes one possible way to address the gaps identified in Section 4 (Figure 7); it is not claimed to be the only, optimal, or evidence-mandated design, and individual components (e.g., specific sensor modalities, specific transformer variants) are included as representative examples of a design direction rather than as requirements justified by the reviewed literature. The schematic illustrates a proposed progression from raw data acquisition at decentralized nodes ($n_{1},n_{2},n_{n}$)—incorporating multi-sensor fusion (RGB, LiDAR, and hyperspectral)—to global model synthesis via federated learning. Central to the proposal is a hybrid CNN-Transformer model combining a MobileNetV2 backbone with Vision and Swin Transformer encoders, integrated through an attention-weighted fusion mechanism. The pipeline further proposes explainability reporting and secure data-exchange modules, culminating in a closed-loop feedback cycle intended to refine the model using field data over time.

In summary, while methodological innovation is evident, persistent gaps in dataset diversity, real-world validation, integration, computational efficiency, explainability, fairness, and scalability limit practical adoption. To provide an overall view of these limitations, Figure 7 summarizes the gaps identified through our critical analysis across the 50 reviewed studies. Altogether, addressing these gaps motivates the conceptual, unvalidated framework illustrated in Figure 8: a proposed direction for future research, not a system that has been built, deployed, or empirically evaluated in this review. The framework in Figure 8 is organized around the four highest frequency gap categories identified in Figure 7: limited dataset diversity (24 studies), lack of real-world validation (13), integration gaps (9), and limited federated/edge AI adoption (6) and proposes one illustrative combination of techniques (multi-sensor fusion, federated learning, closed-loop feedback) that could jointly address them. The specific components shown (e.g., particular sensor modalities, transformer variants, aggregation protocols) are illustrative choices rather than gap-justified requirements, and alternative architectures addressing the same gap categories are equally plausible.

This proposed workflow would originate at a data acquisition and privacy layer, where decentralized nodes ($n_{1},n_{2},n_{n}$) representing municipal, medical, and hazardous waste facilities could apply privacy-preserving federated learning. By processing multi-sensor data  RGB, LiDAR, and hyperspectral fusion  at the edge, such a design is intended to reduce data-sharing/privacy constraints and improve access to rare material signatures; this remains a design proposal, not a tested claim.

The proposed aggregation stage would merge local model updates using standard federated averaging (FedAvg) [78]:

(1) $$W^{\left(t+1\right)}=\sum_{k=1}^{K}\alpha_{k}W_{k}^{\left(t+1\right)},\;\mathrm{where}\;\sum_{k=1}^{K}\alpha_{k}=1$$

where *K* is the number of participating client nodes, $W_{k}^{\left(t+1\right)}$ is the locally updated model weight vector from client *k* after round *t*, and $\alpha_{k}$ is that client’s aggregation weight, conventionally defined as $\alpha_{k}=n_{k}/\sum_{j=1}^{K}n_{j}$, where $n_{k}$ denotes the number of training samples at client *k*. This follows the standard FedAvg formulation [78]; no modification to the aggregation rule itself is proposed here. Encryption and secure aggregation protocols proposed for this stage would protect the confidentiality of transmitted updates, but confidentiality mechanisms alone do not prevent model poisoning: a malicious or corrupted client can still submit a valid-looking but harmful update under encryption. Robustness to poisoning would instead require robust-aggregation rules (e.g. coordinate-wise median or trimmed mean), gradient clipping, anomaly/outlier detection across client updates, or Byzantine-resilient aggregation schemes.

The proposed classification component is a hybrid CNN-Transformer model using a MobileNetV2 backbone for local feature extraction alongside Vision Transformer (ViT) and Swin Transformer encoders for global and shifted-window attention; patch embedding is treated here as the input-tokenization stage of the ViT/Swin branches rather than as an independent third encoder. These streams would be combined via an attention-weighted fusion, with weights ($\alpha_{S}$) determining the fused feature vector:

(2) $$F_{\mathrm{fused}}=\alpha_{M}v_{M}+\alpha_{V}v_{V}+\alpha_{S}v_{S}$$

where $v_{M}$, $v_{V}$, and $v_{S}$ are the feature vectors produced by the MobileNetV2, ViT, and Swin branches respectively, and $\alpha_{M},\alpha_{V},\alpha_{S}$ are learned fusion coefficients optimized during training through an ensemble-based fusion strategy, with $\alpha_{M}+\alpha_{V}+\alpha_{S}=1$ [14]. This fused vector would then be processed through an MLP with GELU activation and a softmax layer to output class probabilities for hazardous, organic, and recyclable materials—again, as a proposed design, not an implemented and evaluated system.

Beyond model output, the proposal includes a reporting layer intended to support operational trust: attribution-based explainability methods (e.g., Grad-CAM, SHAP – to be selected and validated, not merely asserted) alongside standard classification-performance reporting (e.g., a confusion matrix tracking true/false positives per class). We are explicit that a confusion matrix or TP/FP tracking by itself demonstrates predictive performance, not explainability or algorithmic fairness; genuine fairness assessment would require dedicated subgroup/bias analysis (e.g., performance stratified by waste type, region, or lighting/environmental condition), which is not part of the proposal as currently specified and would need to be designed separately. Finally, the pipeline proposes a closed-loop refinement cycle in which field-deployment data and sortation outcomes would feed back to retrain the model over time, intended to link edge-based sensing to downstream material-recovery tracking. We reiterate that Figure 8 in its entirety is a conceptual direction for future research arising from the gaps identified in Section 4, and no component of it has been implemented or empirically validated within the scope of this review.

Although Figure 8 is presented as a research agenda, it should not be read as a ready-to-deploy design. Deploying such a multi-sensor edge system in real municipal settings would face practical and economic barriers that the reviewed studies rarely quantify. First, LiDAR and hyperspectral sensors are typically far costlier and more maintenance-intensive than the ultrasonic, load-cell, and camera hardware used in the reviewed prototypes [13,36,41]; they would also require calibration and protection from dust, moisture, and weather over long service lives, which no reviewed study evaluated. Second, transformer-based and ensemble models already impose high computational and energy costs that hinder edge deployment [16,44,48,59], and adding multi-sensor fusion would increase memory, power, and latency demands further, while the lightweight models that fit constrained devices often sacrifice accuracy [47,57]. Third, reviewed IoT prototypes already report latency, sensor or network failures, and dependence on reliable internet [36,58,67], and federated learning, which requires repeated model-update exchange with a coordinating server, would aggravate these problems. Fourth, federated and closed-loop pipelines need data-governance agreements across municipal, medical, and hazardous-waste facilities, secure-aggregation infrastructure, and skilled staff for maintenance, retraining, and auditing; the cost and latency overhead already reported for blockchain-integrated smart bins [41] suggests that each added system layer carries real costs. No reviewed study evaluates capital cost, operating cost, or payback against existing sorting practice at municipal scale. These barriers are most acute in the low-income and rapidly urbanizing regions identified in Section 1 as most affected, where budgets, power, and connectivity are most constrained. Figure 8 is therefore a long-term research direction; lower-cost camera- and ultrasonic-based edge systems are a more realistic starting point, and any multi-sensor extension requires field-level cost-benefit evaluation before adoption.

## 5. Conclusions

This systematic literature review consolidates evidence from fifty rigorously selected studies on intelligent waste classification and management systems. Rather than offering a single pooled impression of the field, the conclusions below are deliberately stratified by task, metric type, and deployment stage, so that each claim reflects what the underlying evidence actually supports rather than a general assertion. Reported technical performance is high but narrowly evidenced. Most classification and detection studies report accuracies above 90%, with the strongest architectures (hybrid and ensemble models) reaching close to 99%. These figures, however, describe in-domain performance on each study’s own curated test data, and a separate set of studies reporting forecasting or regression outcomes uses different, non-comparable metrics, so no single accuracy figure can be claimed for the field as a whole. Most importantly, only a small minority of the reviewed studies directly compared an AI- or IoT-enabled system against a traditional, non-AI waste-management approach. Claims that these systems outperform conventional practice therefore remain largely unverified and should be treated as an open question rather than an established finding.

Deployment maturity is similarly uneven across the field. Only a minority of studies implemented a physical hardware prototype, and none reported multi-site or long-term, city-scale operation; real-world field testing was reported in only a handful of cases. Maturity also differs by research focus: deep learning-based classification shows the strongest reported accuracy but the weakest integration with sensing or operational systems; IoT-enabled smart-bin architectures show the most direct physical deployment but remain largely reactive rather than adaptive, and are validated mostly at small, single-site scale; predictive-analytics studies show moderate deployment maturity but limited generalizability beyond the city or time period they were trained on; and domain-specific applications such as e-waste, medical, and hazardous-waste systems show the greatest potential impact but the least empirical validation. Statements about scalability or real-world readiness should be understood as reflecting this uneven mix, not a single verdict on “the field.”

Reported sustainability and environmental benefits require the same caution. Most studies assert positive environmental outcomes, such as reduced landfill use or improved recycling accuracy, but these claims are largely narrative rather than empirically measured; only a small number of studies report actual emissions, energy, or lifecycle data. Sustainability benefit should therefore be read as a stated goal of these systems rather than a consistently demonstrated outcome, and claims of practical superiority should be limited to the specific systems and comparisons where they were actually tested.

Beyond these caveats, the field remains structurally fragmented. The lack of standardized datasets, shared evaluation protocols, and interoperable architectures limits comparability across studies, and the continued separation between perception models, sensing hardware, and operational decision-making shows that most existing systems address only part of the overall waste-management pipeline rather than offering an integrated, deployment-ready solution.

This review is itself limited by its reliance on peer-reviewed sources from selected databases, which may exclude relevant grey literature, very recent work, or unpublished industry and municipal deployments. Cluster assignment, gap coding, and deployment-readiness scoring were resolved by reviewer consensus rather than blinded parallel coding, so no formal inter-rater agreement statistic is reported for these judgments. The search strategy was also weighted toward visual classification and detection terminology, which may have limited retrieval completeness for predictive-analytics and sensor-fusion studies relative to classification studies. The vocabulary likewise did not target adjacent solid-waste domains such as mining waste, or sensor-coupled recognition pipelines beyond RGB imaging (e.g., [31,75]), so such work was not systematically retrieved. Finally, the heterogeneity of datasets, metrics, and reporting practices across the reviewed literature limits direct quantitative comparison and should be kept in mind when interpreting the figures above.

Future work should prioritize direct, controlled comparisons against traditional waste-management baselines and multi-site or longitudinal deployment studies that report operating duration, reliability, and environmental robustness, rather than single-site pilot results alone. Developing integrated architectures that combine sensing, perception, and operational analytics within a single framework remains an important direction, as does pairing academic reviews with industry and municipal case studies to better capture real-world adoption. Federated learning, multi-sensor data fusion, edge intelligence, and explainable AI all offer promising paths toward more robust and scalable systems, provided future work evaluates them against the same baseline-comparison and deployment-readiness standards this review found largely missing from the current literature.

## Figures and Tables

**Table 1:** Quality assessment of [14] using our quality appraisal rubric. The study achieved a total score of 88/100 and was rated as Good. Independent evaluations were performed by two reviewers; reviewer identities and task allocation are reported in Appendix B.

SL No.	Quality Assessment Criterion	Yes	No	Other	Score (/10)
1	Focused research question clearly described	✔			10
2	Eligibility criteria for data and methods clearly specified	✔			10
3	Comprehensive literature coverage and comparative analysis	✔			9
4	Independent or comparative validation against baseline/alternative methods		✔		6
5	Dataset quality and representativeness clearly assessed		✔		6
6	Datasets and experimental results clearly reported	✔			10
7	Limitations and potential sources of bias explicitly discussed		✔		7
8	Robustness and generalizability testing (e.g., ablation study, cross-dataset validation, or sensitivity analysis)	✔			10
9	Data and code availability to support reproducibility	✔			10
10	Consistency and alignment with existing literature	✔			10
Total Quality Score					88/100
Quality Rating					Good
Raters: R1: H.Y.A., R2: T.M.					R1, R2

**Table 2:** Comparison of related reviews. Novelty is framed relative to each review’s actual scope rather than as an unqualified claim of comprehensiveness.

Review	Focus	Yr	PRISMA	Scope	Taxo.	Key Gap vs. Our Study
[10]	SLR, 97 studies; ML/DL/hybrid taxonomy; 15+ datasets reviewed	2025	✔	Models and datasets	✔	No IoT hardware, sensing, or deployment-readiness analysis
[23]	Financing methods; circular economy typology	2024	✔	Municipal financing	✔	No AI/technical depth
[24]	AI + IoT for urban waste management	2025	✗	Smart cities	✔	Narrative only; no systematic rigor
[25]	AI in municipal waste management	2025	✔	2010–2024, municipal	✔	Broad scope; not classification-specific
[26]	Life Cycle Assessment in MSW	2025	✔	2003–2023, global LCA	✗	LCA only; no AI/IoT
[27]	ML/DL in MSW management, 69 studies	2025	✔	2018–2024, MSWM	✔	No IoT integration; no clustering
[28]	SLR, 28 studies (56 total); ML/DL taxonomy for plastic waste detection and classification	2024	✔	2000–2023, Plastic waste	✔	Model accuracy focus; lacks sensor-centric system analysis, fusion, edge deployment, and reporting-gap analysis
**Our Study**	**PRISMA SLR of ML, DL, IoT approaches with sensor-centric system analysis**	**2026**	✔	**2020–2025**	✔	**Adds sensing, fusion, edge deployment, and reporting-gap analysis alongside model performance**

**Table 3:** Summary of insight analysis across the 50 reviewed studies, organized by thematic clusters.

Cluster	No. of Studies	Primary Techniques	Main Application	Key Observed Limitations
IoT-Enabled Smart Bin Architectures	14	Ultrasonic, gas, and weight sensors; ESP32; Raspberry Pi	Monitoring, alerts, and automation	Pilot-scale validation, limited waste-category coverage, communication latency, sensor drift/reliability issues, and reduced sensing performance under variable waste conditions (e.g., moisture and heterogeneous materials).
Deep Learning for Visual Waste Recognition	20	CNNs, YOLO, EfficientNet, and hybrid ensemble models	Waste classification and detection	Limited dataset diversity, class imbalance, domain shift between laboratory and real-world environments, and reduced recognition robustness under occlusion, deformation, and complex backgrounds.
Predictive Analytics and Operational Optimization	9	LSTM, XGBoost, and regression models	Bin-fill forecasting, routing, and planning	Dependence on location-specific historical data, limited integration with operational systems, offline validation, and reduced forecasting reliability under dynamic waste-generation patterns.
Domain-Specific and Emerging Frontiers	7	Edge AI, federated learning, and multimodal models	Medical, disaster, and hazardous waste management	Limited application coverage, high computational requirements, insufficient field validation, performance degradation after model compression, and limited cross-site transferability.

**Table 4:** Summary of 50 reviewed studies on municipal solid waste (MSW) classification, showing dominant models, reported top metric value, evaluation metrics, hardware setup, monitoring approach, waste categories, usability, limitations, future improvements, environmental impact of study. Note: The “Reported Top Metric” column reports each study’s own best-performing headline number in the metric that study used (accuracy, precision/recall, mAP, $R^{2}$, correlation, MAPE/RMSE, or a qualitative validation statement, as indicated in the adjacent “Evaluation Metrics” column); values are not directly comparable across rows with different metric types and should not be pooled into a single cross-study statistic without first grouping by task and metric (see Section 4). These values further reflect in-domain performance on each study’s own curated test/validation split and should not be read as evidence of robustness to lighting, occlusion, sensor noise, or geographic/domain transfer unless the study explicitly reports external or cross-domain validation.

Paper ID	Most Dominant Model	Reported Top Metric	Evaluation Metrics	Hardware	Monitoring	Waste Categories	Usability	Limitations	Future	Environment Impact
[13]	ResNet34 (better than AlexNet, VGG16, ResNet50)	95.31%	Accuracy, Box Classification, SUS	ESP8266, ultrasonic + load sensors	Bluetooth + IoT monitoring	Bio and non-bio (5 classes)	Smart home waste system	Few sensors, limited categories, occasional faults	More datasets, sensors, federated learning	Eco-friendly household waste management
[34]	DP-CNN + En-ELM lightweight model	96%	Accuracy, Precision, Recall, F1, AUC	Arduino Mega, webcam, servo motors	GUI monitoring (∼3 FPS)	Bio/non-bio → 9 → 36 classes	Lightweight GUI based system	Rare classes, controlled testing	Edge AI, federated learning, larger datasets	Reduces landfill and improves recycling
[14]	Hybrid Ensemble (ResNet50 + EfficientNetV2 + DenseNet201)	98.29%	Accuracy, Precision, Recall, F1	Multi-GPU training setup	Automated classification	10 waste categories	High-accuracy ensemble classification	Small dataset, class imbalance	Transformer hybrid, IoT integration	Improves recycling efficiency
[35]	CNN predictive waste generation model	$R^{2}$ = 0.999	ARE, RMSE, AARE, $R^{2}$	Cloud ML infrastructure	Predictive monitoring	Population, waste type, geography	Municipal waste planning support	Context-specific model limitations	IoT, GIS, blockchain integration	Improves urban waste planning
[36]	DenseNet121 with CPS-IoT	94.63%	Accuracy, Precision, Recall, F1	ESP32, camera, ultrasonic sensor, servo	IoT bin level monitoring	Paper, plastic, glass, metal, organic, e-waste	Automated smart bin segregation	ThingSpeak delay, small dataset	Solar powered bins, GSM alerts	Reduces landfill and improves recycling
[37]	Deep Belief Network + OCSO optimizer	CC = 0.996	CC, MSE, RMSE	Experimental DL framework	Waste energy prediction	Elemental composition data	Automated HHV estimation	High computation, limited validation	IoT, digital twin integration	Improves waste-to-energy efficiency
[15]	Hybrid MobileNet + EfficientNet + SNN	98%	Accuracy, Precision, Recall, F1, ROC	DL model dataset (3000+ images)	Automated classification	E-waste devices	Scalable recycling system	Small dataset, labeling issues	Larger datasets, robotics integration	Improves e-waste recycling safety
[38]	MobileNet SSD with UAV detection	92%	Precision, Recall, mAP	Drone camera + Raspberry Pi	IoT waste monitoring	Plastic waste types	UAV-based plastic detection	Long training time, terrain issues	More sensors and datasets	Reduces plastic pollution
[39]	ResNet based CNN	98.16%	Accuracy, Precision, Recall, F1	Deep learning framework	Automated classification	10 waste types	Smart city waste sorting	Imbalanced dataset	Lightweight CNN, multimodal sensors	Improves recycling and sustainability
[40]	YOLOv8 + YOLOv5 detection system	96%	Accuracy, Precision, Recall, F1	Raspberry Pi + sensors + camera	Real-time bin monitoring	Overflow and waste status	Smart bin detection system	Low accuracy for wet trash	Edge optimization	Reduces overflow and fuel use
[16]	Enhanced Swin + ConvNeXt with spatial attention	98.97%	Accuracy, Precision, Recall	Lightweight DL model	Real-time classification	Paper, plastic, glass, organic	Efficient real-time garbage classifier	Limited dataset diversity	IoT integration, larger datasets	Reduces pollution and improves recycling
[17]	YOLOv8 object detection model	mAP@0.5 = 0.82	Precision, Recall, mAP	Tesla T4 GPU training (Colab)	Real-time e-waste detection	Electronic components categories	Lightweight detection architecture	Small dataset, orientation issues	Edge deployment, larger datasets	Supports safe e-waste recycling
[41]	CNN with IoT sensors and blockchain	95%	Accuracy, Precision, Recall, F1	Smart bins with camera and sensors	Real-time IoT monitoring	Recyclable vs non-recyclable	Smart city waste management system	Cost, latency, blockchain overhead	Edge AI and lightweight blockchain	Reduces emissions and fuel usage
[42]	VGG16 + CSO optimized Random Forest	85%	Accuracy, AUC, Precision, Recall	Standard ML/DL setup	IoT smart city monitoring	Cardboard, glass, metal, paper, plastic	Hybrid DL-ML classification model	Small dataset, moderate accuracy	Transformer-based hybrid models	Improves recycling systems
[43]	Decision Tree + BPSO optimization	99%	Accuracy, Macro-F1	IoT sensors + cloud database	Real-time overflow alerts	Urban waste collection centers	Mobile alert maintenance system	Single city dataset	Advanced ML and GIS integration	Reduces fuel consumption
[18]	Enhanced ResNet50 feature fusion model	94.13%	Accuracy, speed, parameters	Deep learning training setup	Image classification system	TrashNet dataset categories	Efficient lightweight classification	Limited dataset size	GAN data augmentation, IoT integration	Supports recycling processes
[44]	Dual-stream DenseNet + MaxViT classifier	83.11%	Accuracy, Precision, Recall, mAP	TensorFlow DL training	Industrial waste classification	Plastics, glass, cardboard, containers	Conveyor belt sorting system	High computation requirements	Edge AI optimization	Improves industrial recycling efficiency
[45]	Improved CNN with LeakyReLU + Dropout	93.28%	Accuracy, Precision, Recall, F1	Standard DL training	Household waste classification	Organic vs recyclable	Automated segregation model	Binary classification limitation	Multi-class expansion	Reduces landfill waste
[19]	XGBoost regression model	98% precision	$R^{2}$, RMSE, MSE, MAE	Python ML libraries	Construction waste prediction	Concrete, steel, bricks, wood	Data-driven waste estimation	Limited material categories	Digital twin and hybrid ML models	Supports sustainable construction
[46]	DenseNet201+ MobileNetV2 hybrid (RWC Net)	95.01%	Accuracy, Recall, Specificity, F1	PyTorch DL training	Automated recyclable classification	Cardboard, glass, metal, plastic	Efficient recyclable waste sorting	Limited real-world deployment	IoT integration and localization	Promotes sustainable recycling
[47]	Quantized YOLOv11n-cls(real time classifier)	92.2%	Accuracy, Precision, Recall, F1	NVIDIA A40 GPU training	Household recycling classification	Multiple recycling bin categories	Smart bin + mobile app ecosystem	Region-specific dataset	Federated learning models	Promotes circular economy
[48]	EfficientNetB0 + InceptionV3 + XGBoost hybrid	99.87%	Accuracy, Precision, Recall, AUC	TensorFlow	Solid waste classification	12-class household waste dataset	Highly accurate hybrid classifier	Energy intensive and slow	Model pruning and quantization	Improves recycling efficiency
[49]	ResNetV2 with transfer learning	94.44%	Accuracy, Loss	Raspberry Pi camera + proximity sensors	IoT-enabled smart bins	Biodegradable vs non-biodegradable	Automated bin allocation system	Sensitive to lighting and occlusion	Multi-class expansion and IoT sensor fusion	Reduces landfill dumping
[20]	Modified LSTM forecasting model	90%	Accuracy, Recall	Smart bin with ultrasonic, odor, weight sensors	IoT cloud monitoring	Garbage level and gas monitoring	Alert notifications for sanitation workers	Limited pollutant types and latency	Hybrid forecasting models and larger datasets	Improves smart city sanitation
[50]	Improved ResNet + AEO optimizer + SAE classifier	99.55%	Accuracy, Precision, Recall, AUC	IoT camera sensors + GPU workstation	IoT-based real-time classification	Cardboard, glass, metal, paper, plastic	Automated classification with cloud integration	Small dataset and controlled environment	Real-world validation and larger datasets	Reduces pollution and toxic gases
[51]	VGG16 CNN with grid segmentation	>90%	Accuracy, latency, energy utilization	Raspberry Pi smart bin + robotic arm	IoT-enabled monitoring and alerts	Bio, plastic, glass, metal	Real-time smart waste management	Hardware complexity and limited classes	Lightweight models and transformer hybrids	Supports recycling and smart cities
[52]	Multipath CNN (weakly supervised)	98.33%	Precision, Recall, F1, AUROC	Image dataset + CNN training	Illegal dump detection	Binary waste vs non-waste	Smart city monitoring system	Binary classification limitation	Multi-class detection and IoT integration	Reduces illegal dumping
[53]	AMLWRF (IoT + ML + GA optimization)	96.1%	Accuracy, efficiency, tracking rate	Arduino UNO + ultrasonic sensors	IoT bin status monitoring	Wet vs dry waste categories	Route optimization and recycling management	Controlled environment and limited classes	Federated learning and large-scale validation	Supports circular economy
[21]	LSTM time-series forecasting model	MAPE 1.855	MAE, RMSE, $R^{2}$	IoT smart bins with sensors	Bin fullness prediction	Bin status (fullness level)	Collection planning optimization	Single-city dataset limitation	Multimodal forecasting models	Reduces overflow and fuel usage
[54]	GAN + ARIMA forecasting hybrid model	Validated experimentally	Accuracy charts and statistical analysis	Smart bin with ESP8266, GSM, FPGA	Cloud-based monitoring system	Plastic and metal waste	Smart notifications and resource conversion	GAN instability and impurities	Hybrid AI models and real deployment	Reduces landfill toxins and CO_2_
[55]	EfficientNet-B0 transfer learning model	85%	Accuracy, FLOPS efficiency	Pretrained CNN models	Image-based waste classification	Bio, plastic, glass, metal, paper	Automated recycling classification	Limited dataset and no IoT deployment	CNN-Transformer hybrid models	Supports recycling and sustainability
[56]	CLIP-based Vision Language Model	85.07%	Accuracy	Pretrained VLM models	Plastic resin classification	ABS, HDPE, PS, PVC	Multimodal classification capability	Limited resin types and prompt sensitivity	Fine-tuned multimodal datasets	Supports polymer recycling
[57]	PLDs-CNN + Ridge-ELM	99% (4-class), 96% (12-class)	Accuracy, Precision, Recall, F1, AUC, Confusion Matrix	PC/i9 GPU + Jetson + Arduino Mega, servos, webcam	Real-time GUI with SHAP explainability	Hazardous, household food, recyclable, residual	User-friendly conveyor sorting system	Limited subcategory depth and occlusion handling	Larger datasets, better noise handling, industrial hardware	Promotes recycling and reduces manual sorting errors
[58]	DBESO-optimized KSELM	96.42%	Accuracy, Precision, Recall, Specificity	Arduino Uno + sensors + GSM/GPRS	Real-time IoT sensor monitoring	Paper, cardboard, metal, glass, aluminum foil, general waste	Smart GUI with alerts and scheduling	Sensor/network failures and limited interpretability	Deeper AI integration, end-to-end automation	Reduces fuel usage and emissions
[59]	Dual ensemble CNN + AMIS fusion	96.49%	Accuracy, comparative improvement	CNN architectures + metaheuristic optimization	Disaster waste classification	Mixed debris (construction, hazardous, household)	Post-disaster municipal waste management	Flood-only datasets, high computation	IoT, transformer, GAN, multi-disaster scaling	Minimizes hazards and enhances disaster resilience
[60]	MedBin-Net (lightweight CNN + CSPNeXt + PAFPN)	F1-score: 0.94; Precision: 0.91; Recall: 0.97	Precision, Recall, F1, mAP, FLOPs	RTX 4090 + Intel i7 CPU	GUI with per-object detection and disposal metadata	Medical supplies, bloody objects, radioactive items	End-to-end pipeline for waste detection and disposal	Low performance on small objects	Larger dataset, better small object detection	Safe sorting, reduces health risks
[61]	Extra Trees(best of five tested: AdaBoost, Random Forest, Extra Trees, Decision Tree, CatBoost)	$R^{2}$ = 1.0 (train)/0.97 (test)	$R^{2}$, MSE	None reported	based on periodic chemical analysis (organic %, moisture, ash, pH)	Organic waste (MSW organic fraction, for composting)	Decision-support tool for composting	no real-time or field validation; relies on lab/chemical measurements rather than sensor data	SHAP-based feature-importance analysis identified ash content (%) as the dominant predictor	Supports composting and nutrient reclamation, reducing landfill disposal and associated greenhouse gas emissions
[62]	Response Surface Methodology (RSM) + ML	$R^{2}$ = 0.93	$R^{2}$ scores	Statistical + ML modeling	Hazardous waste stream forecasting	Hazardous, medical, industrial	Policy support for GCC waste management	Limited socioeconomic integration	IoT integration, deep learning, global expansion	Reduces pollution risks
[63]	YOLOv3 + ResNet-50 + XGBoost	98.88%	Accuracy, cost reduction, GHG reduction	Deep learning + IMFO optimization	Waste classification + vehicle routing	Diverse MSW: plastic, glass, metal, organic	Smart city waste management	No IoT, high computation	IoT bins, renewable vehicles, optimized routing	12–18% GHG reduction, 15–20% cost savings
[64]	Custom CNN (495,620 params, 1.27 GFLOPS)	92.5%	Accuracy, Precision, Recall, F1	Low-resource CNN training	Solid waste classification	Aluminum, cardboard, plastic, glass	Real-time classification in smart waste systems	Limited categories, no IoT integration	IoT integration, hybrid models	Supports recycling, reduces pollution
[65]	Light Gradient Boosting Machine (LGBM)	$R^{2}$ = 0.882	$R^{2}$, RMSE, MAPE	VIIRS + WorldPop + FROM-GLC + surveys	Household-level plastic waste prediction	General household plastic	Baseline data for waste management	Small sample, limited generalizability	Larger datasets, temporal forecasting	Supports targeted plastic waste reduction
[66]	SSD MobileNetV2 Quantized (TensorFlow Lite)	>80% average precision	Classifica-tion accuracy, inference time	Raspberry Pi 4 + Pi Camera V2 + Arduino Uno	Real-time fill-level + remote monitoring	Paper, cardboard, glass, metal, plastic	Secure IoT-enabled smart bins	Small dataset, limited classes	Larger datasets, more classes, solar-powered edge devices	Promotes recycling and urban-scale deployment
[67]	MobileNetV2 (PyTorch/fast.ai)	96.57%	Accuracy, Precision, Recall, F1, FPS	Cloud: Intel Xeon + 2x Tesla K40m; Edge: Raspberry Pi 3B+	Web dashboard with real-time status	Paper, glass, metal, plastic, cardboard, trash	Secure, scalable, low-cost edge deployment	Needs reliable internet, TrashNet dataset not fully realistic	Federated learning, robot integration, diverse datasets	Facilitates recycling, reduces landfill
[68]	ResNet50V2 (transfer learning, pre-trained)	98.95%	Accuracy, Precision, Recall, F1, Loss	Google Colab CPU/GPU	Confusion matrix + standard DL evaluation metrics	Paper, plastic, glass, metal, others	Validated for automated waste classification	Dataset-specific, limited class coverage, no real-time deployment	Dataset expansion, ensemble/fusion, hardware prototyping	Increases recycling efficiency, reduces landfill
[69]	ResNeXt-101 (accuracy) + ShuffleNetV2/MobileNetV3 (lightweight)	89.62%	Accuracy, F1, GFLOPs, params	Training: Nvidia GTX 1080 + 64GB RAM; Deployment: cloud→edge	Performance monitoring + federated framework	Medical, e-waste, glass, plastic, cardboard, paper, metal	Edge and cloud adaptable for smart cities	Resource demand, generalization may vary	Experimental FL, RL integration, augmentation	Enables efficient and scalable trash sorting
[70]	Support Vector Machine (SVM)	96%	Accuracy, Mean Squared Error (MSE), Computational Latency (0.67s), Precision, Recall, F1-score	Electronic sensors, IoT-integrated stations, and Edge devices	Real-time IoT monitoring (smart bins), fill-level/gas sensors, and landfill infectious discharge tracking	Organic, Plastic, E-waste, Inert materials, and Industrial/Healthcare waste	Real-time deployment for municipal strategic decision-making and route optimization	Inconsistent historical records in developing regions; lack of technical resources	Hybrid deep learning (CNN/RNN), temporal decomposition, and real-time sensor integration	GHG emissions (CH_4_, CO_2_), leachate contamination of water, and loss of biodiversity
[22]	XGBoost + Random Forest + SVM + Linear Regression	$R^{2}$ = 0.908	$R^{2}$, cross-validation accuracy, confusion matrix	Python (scikit-learn, XGBoost, SciPy)	Residual plots + prediction error histograms	Food/organic, glass, metal, paper, plastic, rubber, wood, yard, special waste	Policy design, resource allocation, urban planning	Data imputation, regional transferability	IoT/AI integration, more granular data, advanced ML	Reduces GHG and landfill use, supports circular economy
[71]	Mask RCNN (DenseNet backbone) + Deep Q-Learning Network (DQLN)	99.3%	Accuracy, Precision, Recall, F1, Confusion Matrix, Loss	Python 3.6.5, standard DL environment	Per-class accuracy, validation metrics	Cardboard, trash, glass, plastic, paper, metal	Scalable for smart city, mobile/edge potential	Dataset scale and diversity, high computational needs	Mobile/real-time deployment, IoT integration, lightweight DRL	Supports automated waste sorting for sustainability
[72]	Custom Convolutional Neural Network (CNN)	Training: ∼98%, Validation: ∼80%	Accuracy, Precision, Recall, F1, Loss curves	Conveyor belt + sensors + camera + PC (TensorFlow/Python)	Real-time feedback, UI with prediction/error	Food waste, leaf, paper, wood, waste, plastic bags, plastic bottles, metal cans	Automated, user-friendly, self-adaptive, robust	Generalization gap, limited deployment/test details, brief hardware info	More data/classes, live deployments, IoT/robotics, advanced optimization	Reduces landfill, increases recycling, adaptable to new waste types
[73]	YOLOv3 (object detection, classification)	YOLOv3: mAP 94.99%, AP up to 99.87%, test accuracy ∼85.3%	AP, mAP, IoU, Precision, Recall, Detection speed (FPS), Missed/-False rates	Windows, Intel i7, 8GB RAM, GTX 1650 Super GPU, Darknet + CUDA/cuDNN	Iterative evaluation at 1,000 iterations, AP/mAP curves	Cardboard, glass, metal, paper, plastic, organic waste	Smart city sorting, automated recycling, real-time deployment	Dataset diversity, occlusion/mixed objects, YOLOv3-tiny accuracy, hardware requirements	More classes, complex scenes, lightweight networks, automation integration	Robust, scalable, automated waste segregation for sustainable cities

**Table 5:** Dominant deep learning models used for waste classification and their reported performance.

Model Family	No. of Studies	Accuracy Range
ResNet Variants	9	94.0–99.0%
EfficientNet Family	6	92.0–99.8%
YOLO (v3–v8)	8	85.0–98.5%
Custom CNN Architectures	7	90.0–98.0%
Hybrid/Ensemble Models	5	96.0–99.9%

Note: Accuracy ranges are computed only from 35 out of 50 studies within each architecture family that report classification accuracy as their primary metric; studies reporting mAP, $R^{2}$, or MAPE/RMSE for the same architecture family are excluded from this table and discussed separately (Section 3.1, Table 6).

**Table 6:** Predictive analytics techniques and operational objectives in intelligent waste management.

Model Type	Application	Reported Performance
LSTM/RNN	Bin fill-level forecasting	RMSE <2, $R^{2}>0.9$
XGBoost	Waste generation prediction	$R^{2}>0.88$
Regression Models	Policy planning	City-dependent accuracy

**Table 7:** Cross-cluster comparison of intelligent waste management research.

Cluster	Accuracy	Deployment	Integration	Sustainability Evaluation
IoT Architectures	–	Low	Partial	Qualitative
Deep Learning Classification	High	Low	Low	Qualitative
Predictive Analytics	High	Medium	Low	Limited
Emerging Frontiers	Variable	Very Low	Conceptual	High (Potential)

Note: Ratings are column-specific and not comparable across columns. Accuracy: High = exclusively ≥90% reported; Variable = wide spread within cluster; “–” = non-comparable metric type. Deployment: Very Low = simulation/conceptual only; Low = lab prototype or single-site pilot; Medium = pilot with extended field validation; High = multi-site/city-scale. Integration: Low = standalone module, no reported linkage to other pipeline stages; Partial = linked to one adjacent stage; Conceptual = proposed but untested. Sustainability Evaluation: Qualitative = impact asserted narratively; Limited = partial quantitative estimate; High (Potential) = sustainability-relevant aim but largely unevaluated empirically.

**Table 8:** Comparative technical synthesis of predictive modeling and optimization frameworks in municipal solid waste management, summarizing algorithms, feature engineering strategies, dataset scale, and quantitative performance metrics to highlight methodological evolution from perception-driven systems to data-driven decision intelligence.

Algorithm/Model	Feature Extraction Techniques	Dataset Size	Key Results
CNN, SVM, MLP, Logistic Regression [35]	Socioeconomic, demographic, geographic variables (20 features)	4341 records (Kaggle)	CNN achieved $R^{2}=0.999$ (train), 0.996 (test/validation); RMSE = 0.53 (validation)
DBN + OCSO [37]	Elemental composition features (ash, C, H, N, O, S, water)	Experimental HHV dataset	CC = 0.996; MSE = 2.342
Decision Tree + Binary PSO [43]	IoT sensor features (fill level, weather, maintenance, etc.; optimized to 3 features)	6954 records; 57 WCCs	>99% accuracy; >99.8% macro-F1
Hybrid CNN (DenseNet201 + MobileNetV2) [46]	Deep visual feature fusion + Score-CAM	2527 images (TrashNet)	95.01% accuracy
1D-CNN, LSTM, GRU, Bi-LSTM [21]	Time-series IoT sensor data (bin fullness, GPS, timestamps)	32 bins (2018–2021)	LSTM best MAPE = 1.855; 1D-CNN lowest RMSE = 1.12
YOLOv3 + ResNet-50 + XGBoost + IMFO routing [63]	Deep visual detection + feature extraction + routing optimization	Real-world urban dataset	98.88% classification; 15–20% cost reduction; 12–18% GHG reduction
LGBM [65]	Remote sensing (VIIRS, WorldPop, FROM-GLC) + socioeconomic household data	200 households (Bali)	$R^{2}=0.882$; MAPE = 12.877
SVM, RF, NB [70]	Socioeconomic + spatial zoning + waste typology features	5-year dataset (2019–2024)	SVM accuracy = 96%; latency = 0.67 s
XGBoost, RF, SVM + Linear Programming [22]	Global MSW indicators (World Bank dataset)	Global dataset	XGBoost $R^{2}=0.908$; 15% operational efficiency gain

**Table 9:** Structured gap–solution–limitation analysis of Cluster 3 studies, mapping research problems to proposed predictive or optimization strategies and identifying recurring challenges in scalability, generalization, real-time integration, and practical deployment.

Research Question/Problem/Gap	Proposed Solution	Limitation
Lack of nonlinear predictive modeling for MSW generation [35]	CNN-based forecasting framework with cross-validation	High computational cost; limited geographic generalization
Costly and harmful calorimetric HHV estimation [37]	DBN + OCSO optimized HHV prediction	Limited real-world validation; elemental-only focus
Reactive waste collection schedules [43]	IoT + ML predictive WCC criticality alerts	Single-city validation; limited waste streams
Moderate accuracy and limited interpretability in recyclable classification [46]	Hybrid CNN + Score-CAM explainability	Small, imbalanced dataset
Poor bin overflow forecasting [21]	LSTM-based time-series forecasting	Single-city IoT dataset
Static routing and high GHG emissions [63]	YOLO + IMFO optimized vehicle routing	High computational demand; limited IoT integration
Scarce household-level plastic waste data [65]	Remote sensing + LGBM modeling	Small sample; region-specific
Lack of emission forecasting capability [70]	SVM-based waste and emission prediction	City-specific; no deep learning integration
Economically unsustainable waste strategies [22]	Multi-model ML + linear programming optimization	Hypothetical optimization assumptions; data gaps

**Table 10:** Domain-specific AI-driven waste management applications across specialized waste streams, highlighting technological innovations, deployment readiness, and sustainability contributions beyond generalized municipal systems.

Paper	Waste Domain	Core AI/Technology	Innovation Type	Deployment Level	Key Performance	Sustainability Contribution	Key Limitation
[15]	E-waste	Hybrid MobileNet + EfficientNet + SNN	Hybrid deep ensemble for hazardous e-waste sorting	Pilot-scale experimental	98% accuracy, AUC=1.00	Reduces toxic exposure, improves recycling efficiency	Small biased dataset; limited rare items
[38]	Plastic waste (riverine)	UAV + IoT + MobileNet SSD (edge AI)	Drone-based edge detection in remote terrains	Field-tested (Bogor river)	92% accuracy	Mitigates plastic and microplastic pollution	Long training time; terrain constraints
[52]	Illegal dumping (urban MSW)	Multipath CNN (weak supervision)	AI-based dump localization for smart cities	Experimental dataset validation	98.33% accuracy	Reduces illegal dumping; supports urban sustainability	Small non-public dataset; binary scope
[56]	C&D Plastic Resin	Vision-Language Models (CLIP family)	Zero-shot and few-shot multimodal classification	Experimental	85.07% (few-shot multimodal)	Enables polymer-specific recycling	Prompt sensitivity; limited resin types
[59]	Disaster waste	Dual-ensemble CNN + AMIS optimization	AI + metaheuristic optimization for crisis waste	Real flood datasets	96.49% accuracy	Enhances disaster resilience and rapid clearance	High computational complexity
[60]	Medical waste	MedBin-Net (lightweight CNN + WHO mapping)	End-to-end detection + WHO-compliant disposal	Experimental + GPU validation	F1 = 0.94	Safer medical disposal; suitable for edge deployment	Limited dataset; excludes some WHO types
[62]	Hazardous waste (GCC)	ML + Response Surface Methodology	Empirical predictive modeling	Policy-level forecasting	$R^{2}=0.93$ (industrial HW)	Supports evidence-based environmental policy	Region-specific; no real-time IoT

Note: Sustainability contribution refers to potential downstream recovery enabled by sorting; physical recovery is not evaluated in the study.

**Table 11:** Emerging technological frontiers in domain-specific waste management, mapping enabling technologies, ecosystem integration potential, scalability maturity, and open research challenges toward next-generation sustainable waste infrastructures.

Emerging Frontier	Representative Papers	Technological Enabler	Ecosystem Integration Potential	Maturity Level	Open Research Gap
Hybrid Deep Ensembles for Hazardous Sorting	[15]	CNN ensembles + SNN + optimization	Smart recycling plants	Medium	Lightweight real-time deployment
Edge AI + UAV Monitoring	[38]	Drones + IoT + edge computing	Remote and riverine waste monitoring	Medium	Robustness in harsh environments
AI-Based Illegal Dump Surveillance	[52]	Weakly supervised CNN	Smart city surveillance systems	Medium	Multi-class expansion; real-world validation
Zero/Few-Shot Multimodal AI	[56]	Vision-Language Models	Data-scarce recycling ecosystems	Emerging	Domain adaptation and prompt automation
Lightweight Edge Medical AI	[60]	Efficient CNN + WHO mapping	Hospital and clinic automation	High (for pilot use)	Small/transparent object detection
Disaster-Aware AI Systems	[59]	Ensemble DL + optimization	Crisis-response waste infrastructure	Emerging	Cross-disaster generalization
ML-Based Hazardous Waste Forecasting	[62]	ML + RSM empirical modeling	Policy and national-level planning	Medium	Real-time IoT integration; socioeconomic modeling

Note: Maturity Level: Emerging = conceptual, simulation, or early experimental stage, with no field deployment; Medium = laboratory-validated or single-site pilot-tested; High = pilot-scale real-world/edge-device validation reported (e.g., field or hospital-site testing).

## Data Availability

This study did not generate new experimental data. All findings are derived from the 50 peer-reviewed publications cited in the reference list, which are publicly accessible through their respective publishers. The database search strings and export records (Appendix A) and the PRISMA screening and exclusion counts (Figure 2) are reported within this manuscript. The review protocol was registered in the Open Science Framework (OSF) and is available at https://doi.org/10.17605/OSF.IO/G9JEK. The quality-appraisal scores for all 50 included studies, the per-study data-extraction records underlying Table 4, the derived Table 3 and Table 5, Table 6, Table 7, Table 8, Table 9, Table 10 and Table 11, and Figure 3, Figure 4, Figure 5, Figure 6 and Figure 7 are available from the corresponding author upon reasonable request.

## Abbreviations

The following abbreviations are used in this manuscript:

IoT	Internet of Things
MSW	Municipal Solid Waste
CNN	Convolutional Neural Network
LSTM	Long Short-Term Memory
CPS	Cyber-Physical System
UAV	Unmanned Aerial Vehicle
PPFL	Privacy-Preserving Federated Learning
XAI	Explainable Artificial Intelligence
SHAP	SHapley Additive exPlanations
VLM	Vision-Language Model
ViT	Vision Transformer
RSM	Response Surface Methodology
GA	Genetic Algorithm
DRL	Deep Reinforcement Learning
DQN	Deep Q-Learning Network
MLP	Multilayer Perceptron
RGB	Red, Green, and Blue
LiDAR	Light Detection and Ranging
GHG	Greenhouse Gas
AUC	Area Under the Curve
mAP	Mean Average Precision

## Appendix A. Database-Specific Search Strategies

The complete database-specific search strategies used for this systematic literature review are reported below. Search syntax was adapted to the respective query language and field structure of each database while maintaining the same conceptual search framework, built around three concept blocks: (1) waste/material terms, (2) computer-vision/deep-learning methods, and (3) automation/application terms.

### Appendix A.1. Scopus

Search date: 18 November 2025

Search string:

TITLE-ABS-KEY(
  ( "waste classification" OR "trash detection" OR "recycling sorting"
    OR "solid waste" )
  AND
  ( "computer vision" OR "deep learning" OR "CNN"
    OR "convolutional neural network" OR "object detection" )
  AND
  ( "automated sorting" OR "intelligent waste management"
    OR "feature extraction" )
)

Applied filters: Document type limited to Article or Conference Paper; Language limited to English; Publication years 2020–2025; Subject area limited to Computer Science, Engineering, and Environmental Science. [The supplementary 15 August 2026 update search, which used an identical query restricted to records added after the primary search date]

Records retrieved: 150

### Appendix A.2. Web of Science Core Collection

Search date: 18 November 2025

Search string:

TS=(
  ( "waste classification" OR "trash detection" OR "recycling sorting"
    OR "solid waste" )
  AND
  ( "computer vision" OR "deep learning" OR "CNN"
    OR "convolutional neural network" OR "object detection" )
  AND
  ( "automated sorting" OR "intelligent waste management"
    OR "feature extraction" )
)

Applied filters: Refined by Document Types (Article, Proceedings Paper); Language (English); Timespan 2020–2025; Web of Science Categories limited to Computer Science and Engineering categories. [The supplementary 15 August 2026 update used the same search query, restricted to records added after the primary search date.]

Records retrieved: 75

### Appendix A.3. IEEE Xplore

Search date: 18 November 2025

Search string:

("All Metadata":"waste classification" OR "All Metadata":"trash detection"
 OR "All Metadata":"recycling sorting" OR "All Metadata":"solid waste")
AND
("All Metadata":"computer vision" OR "All Metadata":"deep learning"
 OR "All Metadata":"CNN" OR "All Metadata":"convolutional neural network"
 OR "All Metadata":"object detection")
AND
("All Metadata":"automated sorting"
 OR "All Metadata":"intelligent waste management"
 OR "All Metadata":"feature extraction")

Applied filters: Content Type limited to Conferences and Journals; Publication Year 2020–2025. [The supplementary 15 August 2026 update used the same search query, restricted to records added after the primary search date.]

Records retrieved: 60

### Appendix A.4. PubMed/MEDLINE

Search date: 18 November 2025

Search string:

("waste classification"[tiab] OR "trash detection"[tiab]
 OR "recycling sorting"[tiab] OR "solid waste"[tiab])
AND
("computer vision"[tiab] OR "deep learning"[tiab] OR "CNN"[tiab]
 OR "convolutional neural network"[tiab] OR "object detection"[tiab])
AND
("automated sorting"[tiab] OR "intelligent waste management"[tiab]
 OR "feature extraction"[tiab])

Applied filters: Publication date 2020/01/01 to 2025/11/18; Article type limited to Journal Article; Language limited to English. [The supplementary 15 August 2026 update used the same search query, restricted to records added after the primary search date.] Records retrieved: 30.

### Appendix A.5. Search Update

The same database-specific search strategies were rerun on 15 August 2026 to identify records published/indexed since the initial search boundary. The update search identified 20 additional records, of which 5 met the eligibility criteria and were included in the final review. These 5 studies, together with 3 studies identified through Google Scholar citation-tracking, account for the 8 studies added to the 42 identified through the primary database search (42 + 3 + 5 = 50).

## Appendix B. Quality-Assessment Rubrics

The quality appraisal of all 50 included studies was performed independently by two reviewers, R1 (H.Y.A.) and R2 (T.M.), each of whom scored every study against the 10-item checklist without reference to the other’s scores. Scores were then compared item-by-item; where the two reviewers assigned different scores to the same item, the discrepancy was resolved by joint discussion and re-inspection of the primary study, and the reconciled score was recorded. No study’s final total was determined by a single reviewer.

Data extraction, cluster coding (assignment of each study to one of the four thematic clusters), gap coding (mapping of reported limitations to the categories shown in Figure 7), and deployment-readiness scoring against the six RQ2 criteria (validation setting, hardware validation, latency, operating duration, communication reliability, and environmental robustness with data modality) were conducted as a consensus exercise rather than by blinded parallel coding: an initial coding was produced for all 50 studies and then reviewed study-by-study against the extracted evidence, with each disputed assignment discussed until agreement was reached and the governing decision rule recorded (see the primary-contribution rule stated in Section 3). Because these subjective codes were reconciled by consensus rather than generated by independent parallel coding, we do not report a chance-corrected inter-rater agreement statistic (e.g., Cohen’s κ) for them, as such a coefficient is not interpretable under a consensus protocol. This is acknowledged as a methodological limitation in Section 5.

Table A1 summarizes the allocation of tasks, and Table 1 presents a representative example of the completed appraisal rubric.

**Table A1:** Allocation of review tasks across the author team. “Independent” denotes parallel assessment carried out separately before reconciliation; “Consensus” denotes joint coding reconciled through discussion, for which a chance-corrected agreement statistic is not reported.

Task	Mode	Resolution Procedure
Title and abstract screening	Consensus	Discussion; record re-read
Full-text eligibility assessment	Consensus	Discussion against stated criteria
Quality appraisal (10-item)	Independent (R1, R2)	Item-by-item comparison; discussion
Data extraction	Extracted and verified against primary studies	Re-check against source article
Cluster coding (4 clusters)	Consensus	Discussion to agreement; rule recorded
Gap coding (spider-chart categories)	Consensus	Discussion to agreement
Deployment-readiness scoring (RQ2)	Consensus	Discussion; criteria fixed a priori
